# Persistent pseudopod splitting is an effective chemotaxis strategy in shallow gradients

**DOI:** 10.1073/pnas.2502368122

**Published:** 2025-05-08

**Authors:** Albert Alonso, Julius B. Kirkegaard, Robert G. Endres

**Affiliations:** ^a^Niels Bohr Institute, University of Copenhagen, Copenhagen 2100, Denmark; ^b^Department of Computer Science, University of Copenhagen, Copenhagen 2100, Denmark; ^c^Department of Life Sciences and Centre for Integrative Systems Biology and Bioinformatics, Imperial College London, London SW7 2AZ, United Kingdom

**Keywords:** chemotaxis, physical limits, reinforcement learning, mechanical intelligence, pseudopods

## Abstract

Single-cell organisms and various cell types use a range of motility modes when following a chemical gradient, but it is unclear which mode is best suited for different gradients. Here, we model directional decision-making in chemotactic amoeboid cells as a stimulus-dependent actin recruitment contest. Pseudopods extending from the cell body compete for a finite actin pool to push the cell in their direction until one pseudopod wins and determines the direction of movement. Our minimal model provides a quantitative understanding of the strategies cells use to reach the physical limit of accurate chemotaxis, aligning with data without explicit gradient sensing or cellular memory for persistence. To generalize our model, we employ reinforcement learning optimization to study the effect of pseudopod suppression, a simple but effective cellular algorithm by which cells can suppress possible directions of movement. Different pseudopod-based chemotaxis strategies emerge naturally depending on the environment and its dynamics. For instance, in static gradients, cells can react faster at the cost of pseudopod accuracy, which is particularly useful in noisy, shallow gradients where it paradoxically increases chemotactic accuracy. In contrast, in dynamics gradients, cells form de novo pseudopods. Overall, our work demonstrates mechanical intelligence for high chemotaxis performance with minimal cellular regulation.

## Significance

Traditional cell-migration models assume that large nucleated cells navigate spatial chemical gradients by sensing differences across their entire length to move toward or away from a source. Our research challenges this view by demonstrating that cells can efficiently navigate using simple, local interactions within pseudopods-protrusions driven by actin polymerization from a finite monomeric actin pool and mutual inhibition. Employing deep reinforcement learning, we investigate how cells may have evolved to optimize their movement by selectively suppressing certain pseudopod growth directions, especially in shallow, difficult-to-sense gradients. This not only advances our understanding of cell behavior in fluctuating environments but also informs the development of bioinspired robots capable of navigation without complex sensory control and feedback mechanisms.

Mechanical intelligence is widespread in nature, by which information processing is deeply embedded in the architecture of living systems (1, 2). For instance, the underlying mechanisms by which cells perform chemotaxis, the directed movement of an organism along a chemical concentration gradient during microbial pathogenesis, wound healing, and immune response, remains a subject of intensive research (3–7). Understanding the tight coupling between sensory cues and locomotion is particularly important, as it reveals effective microscopic-scale navigation strategies that enable cells to sense at fundamental physical limits (4, 5, 8). Here, we focus on studying the role of pseudopod formation as a cellular decision-making mechanism, which represents an important yet not fully understood aspect of cellular navigation (9, 10).

The extension of pseudopods—temporary actin-driven protrusions—allows cells to explore their environment and move directionally in amoeboid locomotion, often in response to chemical cues (1). Experimental evidence shows that pseudopod formation occurs at higher rates in shallow gradients and is more pronounced (11). This behavior may help cells approach the fundamental physical limit of sensing by minimizing the interference caused by movement (9). The ultimate limit is reached when a cell exclusively detects previously unbound ligand molecules, effectively behaving as a ligand-absorbing sensor (9, 12).

Despite the central role of amoeboid cell migration, a comprehensive theoretical framework for pseudopod splitting and its strategic role in accurate chemotaxis remains lacking. Traditional chemotaxis models focus on receptor–ligand interactions on the cell surface, where external signals are processed through complex intracellular signaling pathways to guide directional movement (13, 14). Many assume an “all-knowing” cell that can optimally process signaling information for migration (4, 12). More realistic models exist, which tightly couple signaling, cytoskeletal remodeling, and cell-shaped dynamics (9, 15), but extracting clear mechanistic insights from them remains a challenge.

Here, we model the splitting of pseudopods through the dynamics of actin polymerization, where the competition for a finite resource between the extended pseudopods determines the direction of the next cell movement step. By quantitatively describing intracellular interactions within a simplified, interpretable model, we explore the fundamental principles that may contribute to cellular decision-making during chemotaxis in static and dynamic chemical gradients. Specifically, our results provide insight into the internal mechanisms that cells may employ to overcome stochasticity in sensed measurements. Influenced by experiments, we study simple chemotactic strategies that we later generalize by employing deep reinforcement learning (DRL) (16, 17), allowing us to study how pseudopod suppression may enhance the cell’s ability to efficiently use the environmental information. The found strategies, which result from unconstrained optimization, closely align with cellular behavior, such as the alternation of cells between pseudopod splitting and elongation (9). These findings provide an illustrative framework for understanding the physical principles that may be embedded in living systems, where the cell body acts as an analog machine for both information processing and motility.

## Decision-Making Model

**Fig. 1.**
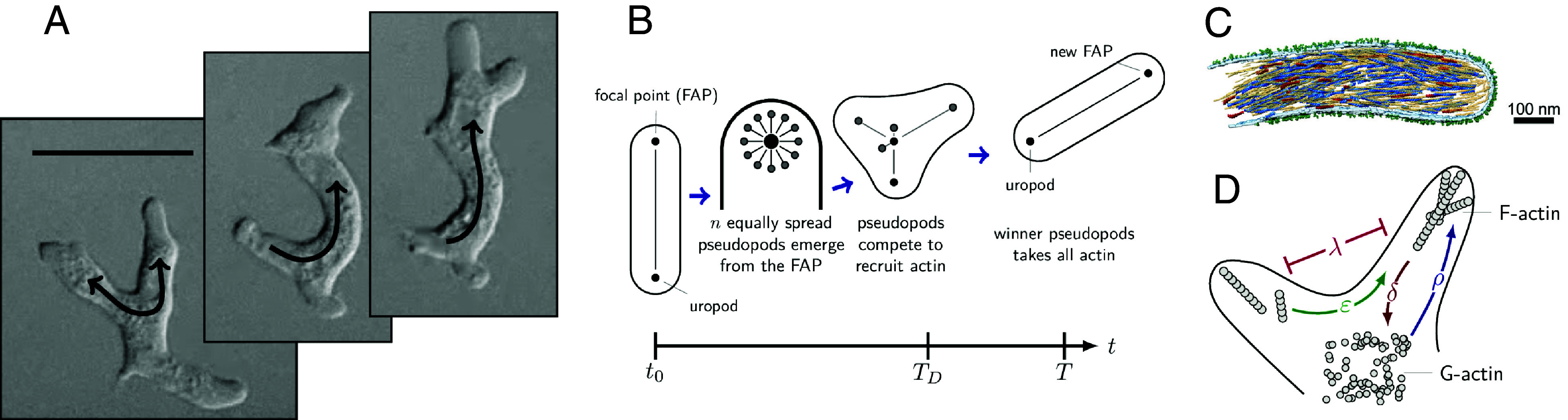
Schematics of the decision-making model and actin dynamics. (*A*) Pseudopod splitting in a *Dictyostelium discoideum* amoeba performing chemotaxis under agar with permission from ref. 10 (rights in the material are owned by a third party). Added arrows indicate likely actin flows, and the scale bar is 20 µm. (*B*) Diagram of the cell morphology during a *splitting event*. Pseudopod formation occurs due to competition during actin recruitment. In this instance, only two pseudopods emerge even though *n* = 12 candidates start the competition. Finally, only one remains after decision-making time *T*_*D*_, altering the cell orientation and advancing its position. The movement step is completed at time *T*. (*C*) Segmentation of actin filaments inside pseudopodia of small platelets with permission from ref. 18 based on high-resolution structural analysis. Actin filaments are in blue, red, and yellow, while receptors are shown in green. Reprinted from ref. 18, which is licensed under CC BY 4.0. (*D*) Schematics of the simplified polymerization of actin into filaments (F-actin) at the internal membrane surface from actin monomers (G-actin), forming pseudopods. The diagram also shows how pseudopods suppress neighbors by redistributing their actin filaments and mutually inhibiting each other’s growth.

In the context of cellular decision-making, pseudopods play an important role that extends far beyond mere movement (9)—we consider them fundamentally coupled with the sensing process. Experimental results show pseudopod formation originates mainly from two distinct mechanisms: splitting from existing pseudopods or by de novo formation (Fig. 1*A*) (11). To simplify this complexity, our minimal model assumes a unified cell body and pseudopod, where the previous pseudopod serves as the origin for new pseudopod growths, which permits us to describe both mechanisms under the same dynamical framework. Similarly, we assume that the new pseudopods grow from the focal adhesion point (FAP) set by the tip of the previous winning pseudopod (Fig. 1*B*). This occurs during a splitting event, which we define as a competitive process between *n* possible directions for the cell to grow pseudopods, where the winning candidate dictates the new cell orientation. We focus our study on *n* = 12 directional options, providing a suitable number of choices for the cell to navigate its environment. In order to win, each candidate attempts to polymerize as many actin filaments (F-actin) as possible from a finite reservoir of actin monomers (G-actin).

### Actin Polymerization.

Pais et al. (19) modeled collective decision-making in honeybee swarms using stochastic differential equations for valued-based decisions with a finite resource to distribute, e.g., swarm members to two or more potential new nest sites. Similarly, our model describes actin polymerization (Fig. 1*C*) in coupled pseudopods with the following system of overdamped Langevin dynamics

[1] $$\begin{array}{c} \frac{dA_{i}}{\mathrm{dt}}=\rho_{i}A_{u}-\delta A_{i}-\lambda A_{i}\overset{¯}{A_{i}}+\varepsilon \left(A_{i}-\overset{¯}{A_{i}}\right)+\eta_{i}\left(t\right), \end{array}$$

where *A*_*i*_ are the cumulative proportion of actin monomers that have been polymerized into filaments at pseudopod $P_{i}$, and $\overset{¯}{A_{i}}$ are the local sum of actin levels of the other pseudopods $\overset{¯}{A_{i}}=\sum_{j\neq i}A_{j}$. Assuming actin mass conservation, we set $A_{u}=1-\sum_{i}A_{i}$ as the uncommitted G-actin proportion inside the cell. This dependence on $\overset{¯}{A_{i}}$ and *A*_*u*_ leads to negative feedback where F-actin grows in a pseudopod at the expense of the others. Similar negative feedback can be found in other models that e.g. describe oscillations of cellular protrusions in Y-junction-shaped microfluidic devices (20). While our model is mechanistically simpler, we allow for external stimuli, cell movement, and more than three cellular protrusions. Our model of stimulus-dependent growth and competition between pseudopods is in line with early proposals that suggests a tight integration of cell migration and chemotaxis (21).

Fluctuations in F-actin in a pseudopod are assumed to arise by propagated cell-external Berg-Purcell noise in chemoattractant concentration (22), which we include by adding white noise *η* with properties

[2] $$\begin{array}{c} \left\langle \eta_{i}\left(t\right)\right\rangle =0,\;\left\langle \eta_{i}\left(t\right)\cdot \eta_{j}\left(t^{\prime }\right)\right\rangle \;=\;\sigma^{2}\cdot c_{i}\cdot \delta \left(t-t^{\prime }\right)\cdot \delta_{\mathrm{ij}}, \end{array}$$

where δ(·) is Dirac’s delta, *δ*_*ij*_ is Kronecker’s delta, and $c_{i}\equiv c\left(x_{i}\right)$ is the concentration level at the tip of pseudopod $P_{i}$. Thus we assume that all relevant noise stems from Poissonian ligand noise, and other sources, such as internal noise due to fluctuations in actin levels, are neglected (but could straightforwardly be included by an additional noise component).

The polymerization rate (*ρ*_*i*_) of actin filaments inside a given pseudopod $P_{i}$ is influenced by the local concentration of chemoattractant, which activates well-known signaling pathways that lead to increased actin polymerization (11). Thus, we model the rate of G-actin recruited by a pseudopod as proportional to the signaling activity of the receptors in the pseudopod. This activity can be defined as the Boltzmann probability *P*_*on*_ of a representative receptor to be in the on (active) state (23). When multiplied by the default rate *ρ*_0_, we obtain

[3] $$\begin{array}{c} \rho_{i}=\rho_{0}\cdot P_{\mathrm{on}}=\frac{\rho_{0}}{1+e^{\Delta F_{i}}}, \end{array}$$

where we linearized the change in free energy for small ligand concentration changes, i.e., $\Delta F_{i}\approx -\kappa_{c}\left(c_{i}-c_{0}\right)$, with *c*_*i*_ and *c*_0_ being the current concentration value at the tip of the pseudopod and its initial value at the FAP, respectively. Hence, the change in free energy contains an indirect local temporal comparison in line with earlier proposals (21). Note that in an environment without a chemoattractant gradient, i.e., at constant concentration, all pseudopods have the same intrinsic polymerization rate set by $\rho =\rho_{0}/2$, leading to random walk behavior in presence of noise.

F-actin constantly undergoes treadmilling, where individual monomers are removed from one end to be added at the other end of the polymer (Fig. 1*D*). We include this by adding a depolymerization rate (*δ*). In our case, however, once the monomer has left the filament, we consider it to be returning to the uncommitted actin pool and, thus, potentially being reused by other pseudopods. Furthermore, pseudopods may inhibit each other by sequestering shared resources and signaling crosstalk (24). Hence, we also include a cross-inhibition (*λ*) term, where the size of the rival candidates induce depolymerization (19).

Finally, the actin exchange rate (*ε*) represents the transfer of actin between pseudopods (Fig. 1*D*), as it has been observed that cells can redistribute actin to prioritize certain directions (25, 26). This term results in a commitment to the winner behavior where the cell follows the largest pseudopod the moment it has grown enough to collapse the other candidates back to the focal adhesion point. The *ε*-term goes beyond the bee model in which a nest site is simply declared the winner when supported by a threshold number of bees (19).

Taken together, our model depends on six parameters (*ρ*_0_, *κ*_*c*_, *δ*, *λ*, *ϵ*, and *σ*). We work in nondimensional units (*Materials and Methods*), where these take values of O(1), but our results are robust to perturbations in their values. An estimation of the values of the parameters based on available data can be found in *SI Appendix*, *Supplement* 2.

### Pseudopod Growth.

To model pseudopod growth, we assume actin to be the sole driver of membrane expansion, thereby linking sensing mechanisms to cellular motility. Hence, by focusing on its intrinsic coupling, we ignore some known effects of membrane mechanics on cellular motility, such as membrane tension, substrate interaction, or surface curvature. Due to the high fluctuations in the actin dynamics at elevated chemoattractant concentrations, we model pseudopod length as a timed-average linear response of F-actin levels,

[4] $$\begin{array}{c} \ell_{i}\left(t\right)\;=\;L\int_{t-1}^{t}A_{i}\left(t^{\prime }\right)dt^{\prime }, \end{array}$$

such that the total dimensionless length of the cell is conserved, i.e., $L\;=\;\ell_{u}+\sum_{i}^{n}\ell_{i}$, where *ℓ*_*u*_ is the uropod length set by the uncommitted G-actin pool (*A*_*u*_), with the time for the linear filter used as the characteristic timescale for the dynamics (see *Materials and Methods* and *SI Appendix*, *Supplements* 2 and 8 for further details).

### Decision Time.

At the beginning of a splitting event, we consider all the motility-associated actin to be unpolymerized $A_{i}\left(t_{0}\right)=0$ for all i∈{1,…,n}, and $A_{u}\left(t_{0}\right)=1$. The event ends at *t* = *T* when one candidate has gathered most of the actin ($A_{i}\approx 1$). However, the duration of the event contains both the decision-making process and the final growth of the winning pseudopod until it takes all remaining actin. Since we are interested in the decision time *T*_*D*_, we define it as the time beyond which the length of the winning pseudopod *i* remains larger than the summed lengths of all remaining candidate pseudopods, i.e.

[5] $$\begin{array}{c} T_{D}=\min t\;\;\;\text{s.t.}\;\ell_{i}\left(s\right)\geq \sum_{j\neq i}\ell_{j}\left(s\right)\;\forall s>t. \end{array}$$

### Concentration Profile.

To simulate chemotactic environments, we assume a linear gradient of concentration profile similar to those observed in chemotactic chambers (9). We treat gradient magnitude (*g*_*x*_) and background concentration (*c*_0_) as freely varying initial conditions to investigate their impact on cell decision-making processes. Note that these are defined with unitless variables as described in *Materials and Methods*.

## Results

### Effect of Chemoattractant on Actin Dynamics.

**Fig. 2.**
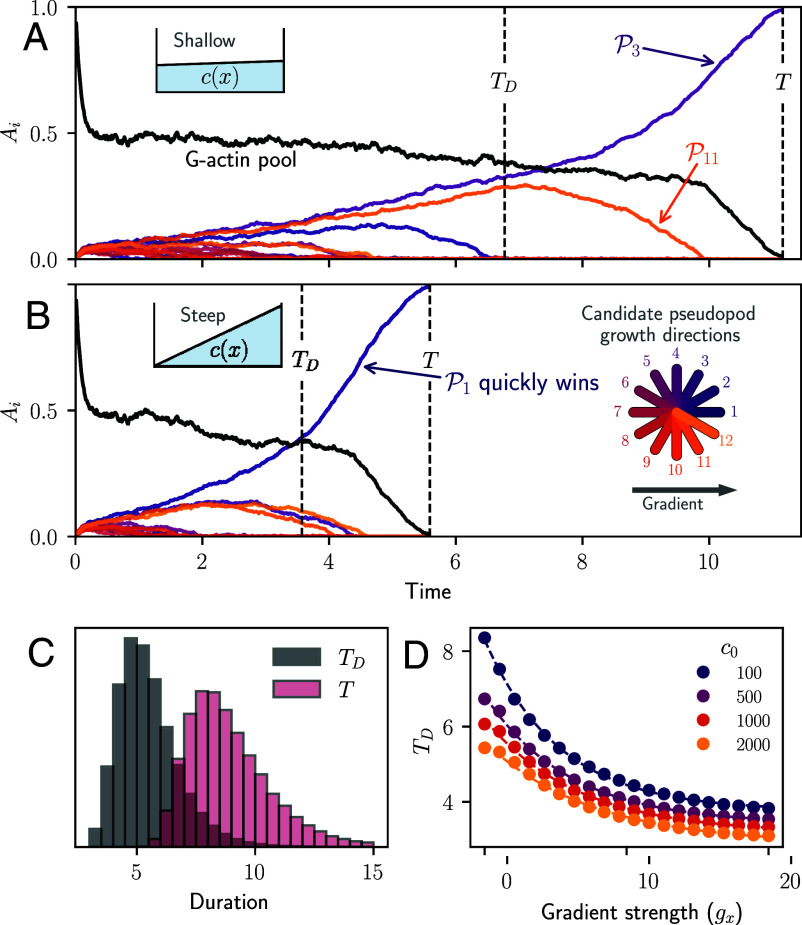
Actin and pseudopods dynamics during decision-making. (*A* and *B*) Sample trajectories of F-actin levels of each of the *n* = 12 candidate directions of the cell, shown in the circular diagram in (*B*), in a linear concentration profile in a shallow (*A*: $g_{x}=0.01$, $c_{0}=10^{3}$) and a steep (*B*: $g_{x}=10$, $c_{0}=10^{3}$) gradient environment, respectively. The proportion of uncommitted G-actin is shown as a black line. The decision time *T*_*D*_, set by Eq. **5**, and the event duration *T* are marked with a dashed vertical lines. (*C*) Duration distributions of event time *T* and decision time *T*_*D*_ for $c_{0}=10^{3}$ and various gradient strengths $g_{x}\in \left(0,10\right)$. (*D*) Decision time as a function of the chemoattractant gradient for different noise levels set by the concentration value. The dashed line corresponds to a fit by $T_{D}=A\cdot \exp\left(-a\cdot g_{x}\right)+T_{D}^{*}$, where $T_{D}^{*}$ is the lowest possible decision time, dependent on the background concentration (*c*_0_).

We evaluate actin dynamics during splitting events under different concentration profiles (Fig. 2). Initially, monomers are polymerized equally by all candidate pseudopods, quickly depleting the G-actin pool (*A*_*u*_) until competition dynamics take over (Fig. 2 *A* and *B*). In shallow gradients (Fig. 2*A*), the candidate that becomes the next pseudopod is randomly decided by the fluctuations during the competition, matching experimental observations that there are more protrusions causing the cells to be more elongated in shallow gradients. When the signal is strong (steep profile), the candidate that aligns with the gradient quickly suppresses the others (Fig. 2*B*). Interestingly, while the duration of the competition is set by the winner candidate polymerizing all the actin (*T*), the decision-making (*T*_*D*_), as defined by Eq. **5**, only accounts for roughly half the event duration (Fig. 2*C*). Decision time decreases exponentially with gradient strength (*g*_*x*_), leading to faster decisions when the signal is strong (Fig. 2*D*). Further, more noise (as set by *c*_0_) leads to faster decisions, but at the cost of accuracy (see *SI Appendix*, *Supplement* 4 for details).

### Emergence of Response Scaling.

**Fig. 3.**
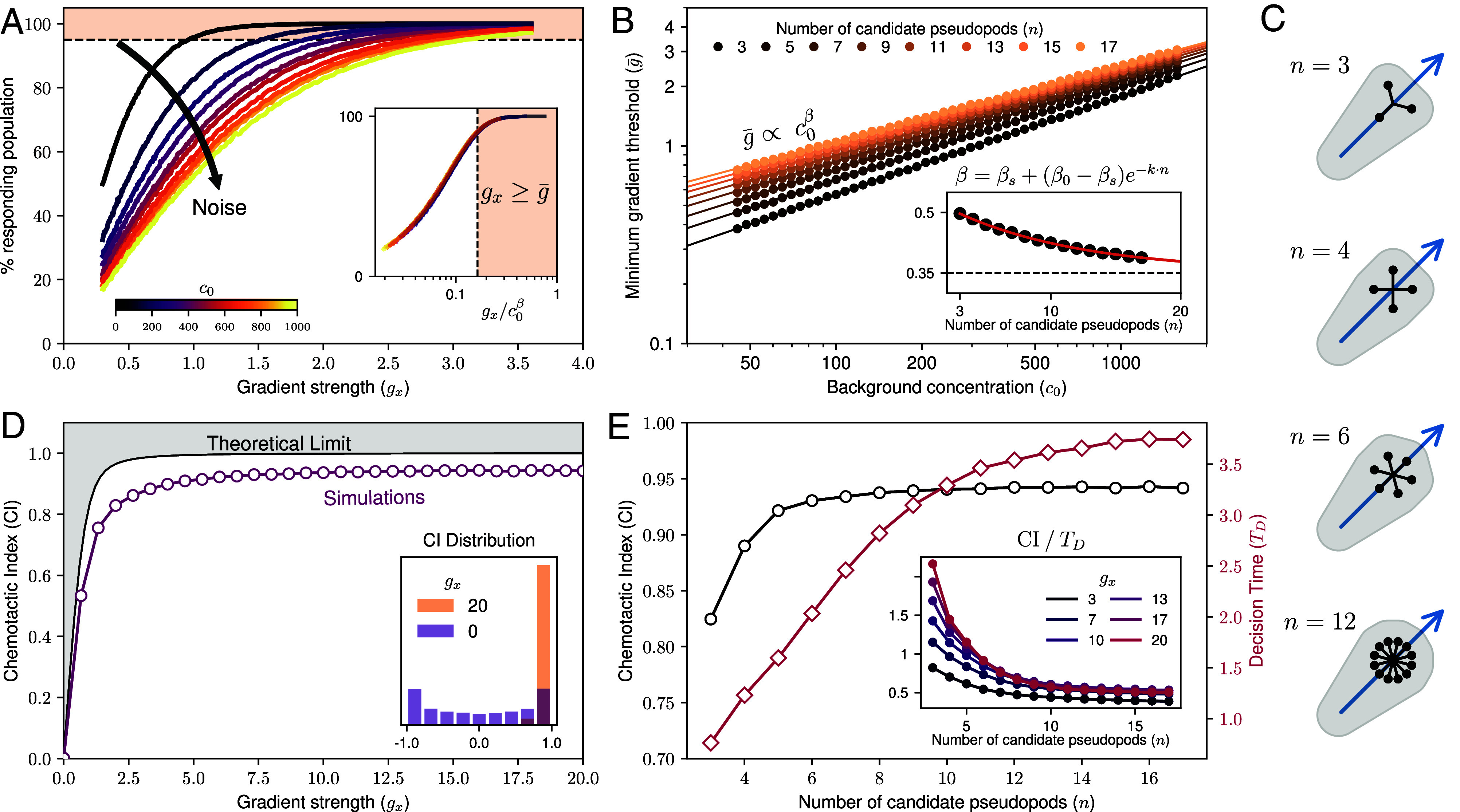
Chemotactic response and speed–accuracy tradeoff. (*A*) Proportion of responding cells, with *n* = 12 candidates, as a function of the gradient signal after performing a splitting event (decision), resulting in movement up the gradient. The response is quantified by the fraction of $10^{4}$ independent splitting events, leading cells to regions of higher concentration, i.e., $c\left(x_{T}\right)>c\left(x_{0}\right)$. Each line represents a different background concentration, illustrating how responses scale based on noise strength. The *Inset* shows a collapse of these responses onto a single curve when plotted against $g_{x}/c_{0}^{\beta }$, with *β* = 0.4. The dashed line and colored area indicate the set response threshold (95%) beyond which cells are defined as performing chemotaxis. In the *Inset*, the gradient ($\overset{¯}{g}$) can be shown as a single vertical line. (*B*) Scaling of the minimum gradient strength $\left(\overset{¯}{g}\right)$ required to achieve >95% responding population. Results are shown for different numbers of candidate pseudopods, which are visualized in (*C*). The *Inset* depicts the fitted *β* values obtained by modeling the simulation results as $\overset{¯}{g}\propto c_{0}^{\beta }$. The red line represents a saturating exponential fit: $\beta =\beta_{s}+\left(\beta_{0}-\beta_{s}\right)\exp\left(-kn\right)$, where *β*_*s*_ is the asymptotic value as n→∞, yielding $\beta_{s}=0.35$. (*C*) Schematic diagrams of candidate pseudopod configurations for various *n*. For *n* = 3, the configuration resembles a Y shape, while *n* = 4 forms an X shape. For higher *n*, pseudopods are distributed equidistantly, with one pointing forward. The blue arrow indicates the cell’s orientation. (*D*) Accuracy of cell alignment with the gradient, measured by the chemotactic index (CI). Simulation results at $c_{0}=500$ are compared with the theoretical limit of a perfectly absorbing cell, as determined by Eq. **8**. This limit is based on prior findings from ref. 12. The *Inset* illustrates the distribution of alignment angles at low and high gradient strengths, showing bimodal peaks at $g_{x}=0$ caused by the nonlinear transformation of random orientations, e.g., cos(u), where u∼U(0,2π). (*E*) CI (black) and mean decision time *T*_*D*_ (red) as functions of the number of candidate pseudopods at $g_{x}=20$. The *Inset* shows the rate of alignment for various gradient strengths (*g*_*x*_) as a function of the number of candidates (n).

We examine the cell’s response to a chemoattractant gradient by measuring the fraction of times in which cells move up the gradient after a splitting event. We scale this fraction to range from 0 (random walk) to 100% (all cell decisions result in movement up the gradient). The response highly depends on both the background concentration (noise) and the gradient signal (Fig. 3*A*), matching qualitatively the nontrivial response in experiments of *Dictyostelium discoideum* reported in ref. 27. Notably, responses can be uniquely characterized by the scaled quantity $g_{x}/c_{0}^{\beta }$, where *β* = 0.4 for *n* = 12 (Fig. 3*A*, *Inset*). This behavior echoes the Weber–Fechner law, which describes a power-law relationship between stimulus strength and background concentration (*β* = 1 for Weber’s law). Here, however, the scaling emerges without relying on logarithmic transformations (4, 23). Instead, local temporal comparisons of concentrations between the initial and current value at the candidate tips enable cells to perform effective gradient sensing simply as a result of competition. To study this scaling, we focus on the minimum stimulus threshold $(\overset{¯}{g}$) for cells to unequivocally perform chemotaxis, which we define as the gradient from which the response is above 95%.

### Competition Regulates Sensing Noise.

Unlike Weber’s law, our minimal stimulus scaling yields an exponent *β* ≠ 1. To understand this scaling, we analyze its dependence on *n*, starting with the intuitive case of *n* = 2. In this scenario, the winning candidate is set by the difference in polymerization rate, which can be approximated as proportional to signal (gradient), and the sensing noise, scaling as the square root of the concentration. This gives *β* = 0.5, equivalent to the signal-to-noise ratio (SNR),

[6] $$\begin{array}{c} \mathrm{SNR}=\frac{g_{x}^{2}}{c_{0}}, \end{array}$$

as also derived in the theory of receptor signaling by Ueda and Shibata (28). Fitting simulation results to the power law $\overset{¯}{g}\propto c_{0}^{\beta }$ (Fig. 3*B*) confirms that the minimal stimulus $(\overset{¯}{g}$) scales as *β* = 0.5 for this intuitive case (Y-shaped cell shown in Fig. 3*C* for *n* = 3). Interestingly, increasing *n* monotonically reduces *β*, as shown in Fig. 3*B*, *Inset*. A saturating exponential fit finds the limit of $\beta_{s}\approx 0.35$, closely matching experimental results by Van Haastert (27). Thus, increasing the number of potential directions leads to better adaptation to increases in noise. However, for the simulated noise levels, the threshold stimulus for chemotaxis is always lower for fewer candidate directions. This difference in scaling suggests a crossing at $c_{0}\approx 10^{4}$, beyond which many candidate directions would result in a lower threshold, potentially explaining the experimentally reported *β* ≈ 0.35 value (27). Intuitively, at high concentration values (high noise), cell-surface receptors send enough signals to promote actin polymerization on many different membrane regions, akin to high *n* in our mechanistic model. Experiments could confirm the different scaling regimes of the threshold stimulus response depending on the background concentration.

### Pseudopod Competition Leads to Indirect Gradient Sensing.

A commonly used measurable observable for chemotactic performance is the chemotactic index, which was previously calculated using the physical limits of sensing (12). The probability of estimating the gradient of a concentration by a perfectly ligand-absorbing cell (to avoid the noise from rebinding) is

[7] $$\begin{array}{c} P\left({\hat{g}}_{x},{\hat{g}}_{y}\right)=\frac{1}{2\pi \sigma_{g}^{2}}\exp\left[\frac{-{\left({\hat{g}}_{x}-g_{x}\right)}^{2}-{\left({\hat{g}}_{y}-g_{y}\right)}^{2}}{2\sigma_{g}^{2}}\right], \end{array}$$

where $\left({\hat{g}}_{x},{\hat{g}}_{y}\right)$ is the estimated gradient, and the real one is given by $\nabla c=(g_{x},g_{y})$, with the uncertainty of the measurement being $\sigma_{g}^{2}=c_{0}/12\pi DT$ (with cell size *a* = 1 in our unitless parameters). Here, *T* and *D* are the time the cell takes to measure the gradient and the diffusion constant of the chemoattractant, respectively. From Eq. **7** and assuming a linear gradient on *x* such that $\nabla c\;=\;(g_{x},0)$, one obtains the expected cosine similarity of the gradient and direction of movement. This chemotactic index is given by

[8] $$\begin{array}{c} \;\text{CI}=\left\langle \cos\left(\theta \right)\right\rangle =\sqrt{\frac{\pi z}{2}}e^{-z}\left[I_{0}\left(z\right)+I_{1}\left(z\right)\right], \end{array}$$

where z=3πk·SNR, *θ* is the orientation of the cell, and *I*_0_ and *I*_1_ are the zeroth- and first-order modified Bessel functions, respectively. The combination k=DT can be thought of as a single fitting parameter which was set by previous literature to match experimental observations (12). During measurement time, it is assumed that the cell processes the measurements by averaging their positional information before making a decision. In our model (Fig. 3*D*) this processing emerges directly from the competition between pseudopod candidates. Initially, during the splitting event, all candidates on the FAP have equal polymerization rates. As pseudopods grow, feedback amplifies those aligning with the gradient direction. However, fluctuations can disrupt this process: A candidate moving slightly ahead up the gradient may reach a higher concentration, leading it to grow more rapidly and consequently suppress its neighbors. Even for strong signals, this stochastic dominance by a leading candidate saturates the decision accuracy to a CI ≈ 0.9, as shown in Fig. 3*D*. Interestingly, this lower saturation, approximately 10% below perfect chemotaxis, is also observed in experiments (12, 29).

### Pseudopod Competition Reveals Speed–Accuracy Trade-Off.

Due to the pseudopods’ fixed orientations, the larger the number of candidates, the more likely a pseudopod will align perfectly with the gradient direction. As just mentioned, the proximity between candidate pseudopods saturates this accuracy. Thus, we compare the resulting alignment in a steep gradient ($g_{x}=20$) for an increasing amount of candidates (Fig. 3*E*) and find that CI increases with *n* up to a saturation point at *n* ≈ 6, from which it remains constant. Curiously, when estimating the average decision time $\left\langle T_{D}\right\rangle$, we also observe a monotonic increase, pointing toward a diminishing return in the number of candidates in terms of efficiency. By evaluating the rate of alignment ($\mathrm{CI}/T_{D}$), we observe a monotonic decreasing efficiency with the number of candidates, which clearly indicates that the lower the number of candidates, the more efficient the decision-making (despite a decrease in absolute gradient estimation accuracy).

### Chemotaxis Trajectories Depend on SNR.

**Fig. 4.**
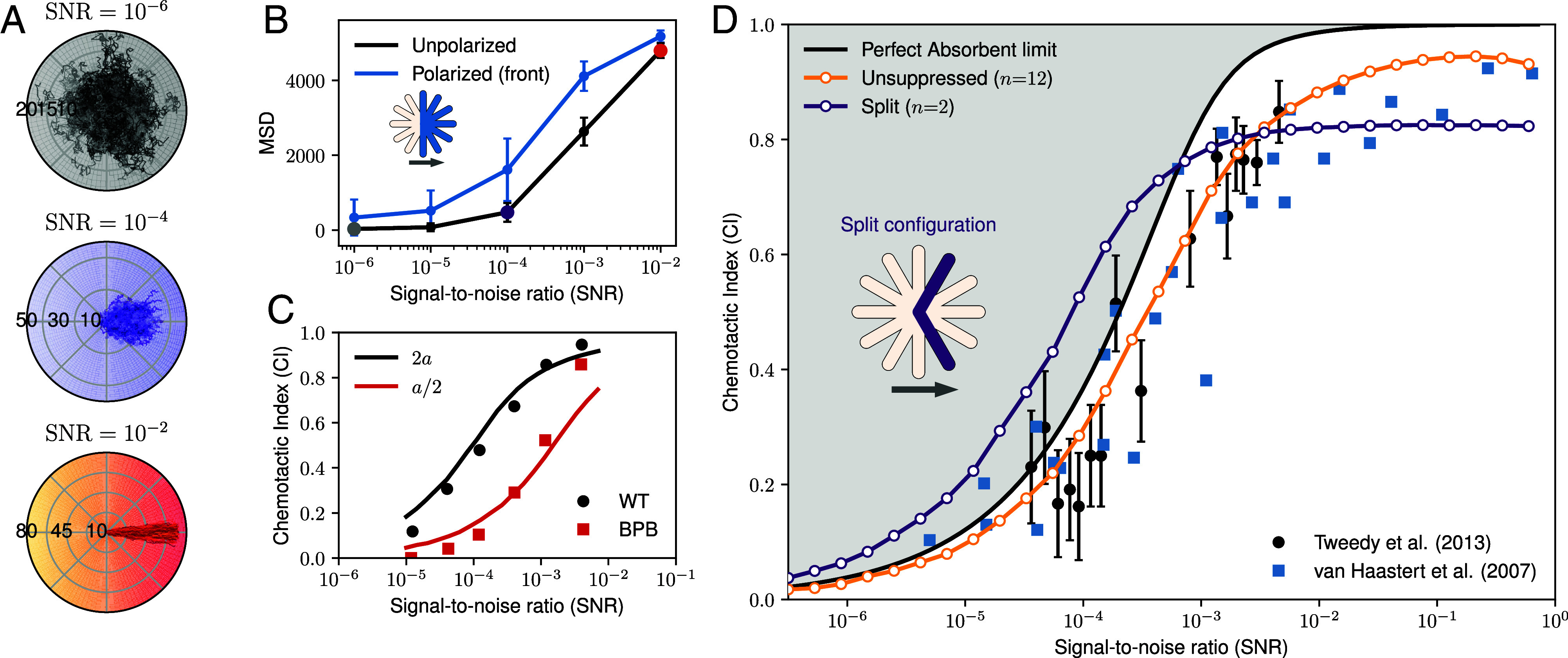
Chemotaxis performance depends on SNR. (*A*) Sample trajectories ($3\times 10^{2}$) from our chemotaxis model, consisting of 15 splitting events, at three different signal-to-noise ratio (SNR) levels with $c_{0}=500$ fixed. (*B*) Mean square displacement (MSD) of $10^{4}$ unpolarized cell trajectories from (*A*) at various SNR levels, color-coded accordingly (dots). For comparison, the MSD of a polarized configuration—with only frontal candidates activated as illustrated in the diagram where blue means activated ($\rho_{0}=1$) and the backfacing candidates are suppressed ($\rho_{0}=0$)—is shown in blue. (*C*) Experimental data from wild-type (black) and p-bromophenacyl bromide (BPB)-treated (red) cells, compared to model predictions at varying length scales. Experimental results are sourced from refs. 9 and 30. (*D*) Chemotactic index (CI) as a function of SNR, plotted on a $\log_{10}$ scale. The theoretical physical limit for a static spherical ligand absorber, derived from Eq. **8** in ref. 12, is fitted to experimental data from refs. 9 and 31. This analysis omits the 0.9 adjustment previously used to account for lower saturation in experimental results. The yellow curve represents the CI of $10^{4}$ independent trajectories, calculated after each splitting event (simulating 15 consecutive splitting events for *n* = 12). The purple line corresponds to the split configuration, where only candidates $P_{3}$ and $P_{11}$ are activated (*n* = 2), as illustrated in the accompanying diagram (*Inset*) where colored means activated ($\rho_{0}=1$) whereas the rest are suppressed ($\rho_{0}=0$). The arrow indicates the facing direction of the cell. For comparison, we show the scaling of an “all-knowing” cell, which infers the gradient from the measurement of its receptors and their known locations, in *SI Appendix*, Fig. S2.

The dynamics of splitting events move the cell body in its chosen direction. When completed, the winning pseudopod becomes the cell body in a new location with a new orientation, ready to start another event. Consequently, in our model, chemotaxis emerges as a sequence of consecutive pseudopod-splitting events. Similar to the chemotactic response, the resulting ensemble displacement is strongly affected by the SNR (Fig. 4*A*), as the spread of the cell during the trajectory is highly correlated with the accuracy of each individual decision. At low SNR, the resulting trajectories converge to a random walk, whereas, at high values, the cells show strong persistence in moving up the gradient. Similarly, polarizing the cell pseudopods (suppressing backfacing candidates) results in higher mean squared displacement (MSD) after the same amount of splitting events (Fig. 4*B*).

Previous work has shown that chemotactic performance can be highly affected by the cell’s ability to create protrusions due to drug treatment (9). We can mimic this behavior in our model by changing the length scale (a), which sets the pseudopod size. This simple change allows us to reproduce the experimental observations without any other modifications to our model (Fig. 4*C*).

### Pseudopod Suppression Enhances Chemotactic Efficiency.

Until now, unless explicitly stated otherwise, we assumed that the cell has *n* = 12 evenly spaced pseudopod candidates, capable of polymerizing actin and sufficient for the cell to accurately choose the right direction. However, fewer candidates can drastically improve efficiency during decision-making (Fig. 3*E*). Experimental observations show that the angle between pseudopods is affected by the chemoattractant gradient shallowness (11, 13). Based on these, we suppress, by setting $\rho_{0}=0$, all pseudopods candidates except $P_{3}$ and $P_{11}$, which grow at angles $\varphi =\pm 60^{{}^{\circ}}$ relative to the cell movement orientation, close to the experimentally observed mean value of $55^{{}^{\circ}}$ (with considerable variability) (13, 32). This *Y* shape is also referred to as the *split* configuration.

In Fig. 4*D*, we compare the chemotactic index of our unsuppressed cell, i.e., with *n* = 12 possible directions of movement (yellow line), with those from experiments (symbols). To quantify the alignment of trajectories, we calculate the chemotactic index as the average of the cosine of the cell orientation, CI=⟨cos(θ)⟩ for binned values of SNR. Interestingly, there is notably excellent agreement between our model and the data. While the unsuppressed cell performs below the fitted fundamental limit of instantaneous gradient estimation (similar to Fig. 3*D*), experimental values can lie above it. Remarkably, the split configuration performs above the limit at low SNR, when the gradient is shallow and difficult to infer, displaying higher robustness against noise compared to the unsuppressed one. Nevertheless, as the quality of the signal increases, the cell with the wider range of options performs better, as the two pseudopods of the split configurations are unlikely to align with the actual gradient. This cross-over in CI can be seen in the data of cell morphology, where amoeba use pseudopod splitting at small SNR and a broad-front polarization at high SNR (9).

In previous work (4), we observed the advantage of gradual orientation updates instead of full turns when optimizing a fully unconstrained spatial policy using DRL at the fundamental limits of ligand sensing. This confirms that relying on persistence is an effective strategy when navigating shallow gradients. In a similar fashion, we ask what DRL predicts about the ideal number of pseudopods and their orientation?

### Optimal Pseudopod Suppression Policy.

Having observed a clear advantage in suppressing possible directions, we explore the possibility of an SNR-dependent optimal configuration in which the cell may have learned to exploit suppressing certain directions to enhance its chemotactic performance. Here, we optimize a mapping function $p_{\theta }:\;R\to {\left[0,1\right]}^{n}$ that indicates the probability of activating candidate *i* by setting their default polymerization rate to either active ($\rho_{0}=1$) or suppressed ($\rho_{0}=0$). We rely on DRL, specifically, proximal policy optimization (PPO) (17), to locate the optimal suppression policy (*p*_*θ*_), here constructed as an artificial feed-forward neural network, given the environment’s state, set by the SNR at the cell location at the start of the splitting event (see *Materials and Methods* for technical details).

**Fig. 5.**
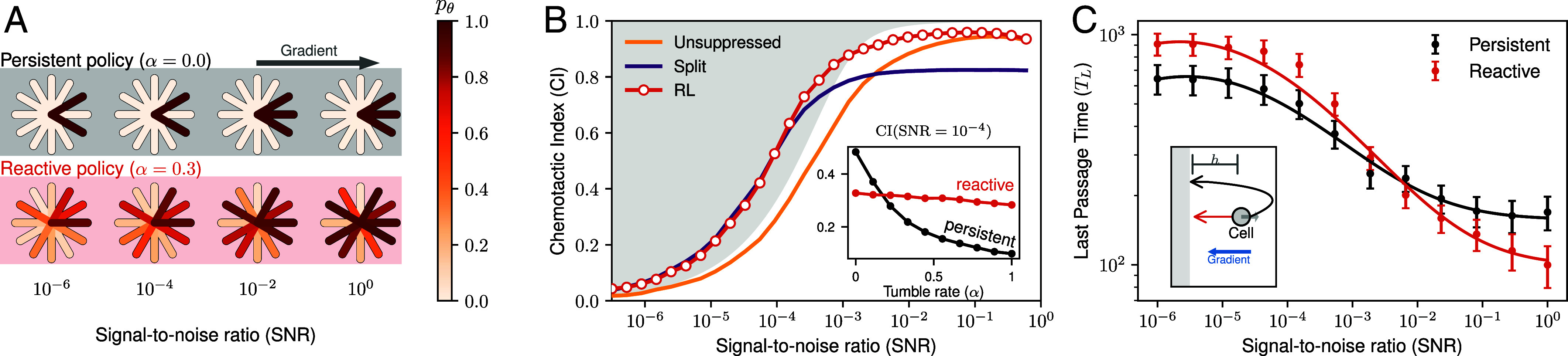
Optimal pseudopod suppression strategy. (*A*) Cell diagrams illustrating the activation probability *p*_*θ*_ for 12 pseudopod candidates at various SNR levels. Two policies are compared using reinforcement learning (RL): a persistent policy, trained in static environments (*α* = 0), and a reactive policy, trained in environments with stochastic mid-trajectory gradient reorientations (*α* = 0.3). Here, *α* sets the probability of randomly changing the gradient direction (see *Materials and Methods* for details). (*B*) Evaluation of the persistent policy (*p*_*θ*_) using proximal policy optimization (PPO), measuring how effectively pseudopods align with the gradient. These results are compared to those in Fig. 4*C*. The *Inset* demonstrates the performance of both the persistent and reactive policies at $\mathrm{SNR}=10^{-4}$, under simulations with increasing rates of random changes in gradient reorientation. (*C*) Time required for a cell to reorient after a sudden reversal of the gradient direction, as a function of SNR, for both trained policies. The accompanying diagrams showcase the experimental setup, where the cell (indicated by the gray arrow) is initially facing *Right*, while the gradient is directed to the *Left* (blue arrow). The reactive policy can instantly switch to reach the gray zone (red arrow), located at a distance *h* = 10, while the persistent policy relies on smaller reorientations, leading to a characteristic U-turn.

The ability of a cell to react and adapt to sudden changes in the environment is another criterion of successful chemotaxis. Specifically, Aquino et al. (33) investigated how microorganisms respond to abrupt changes in the chemoattractant direction, demonstrating that cells can adapt when the direction is suddenly reversed, at least in steep gradients. We introduce the possibility, set by the rate *α*, for the gradient direction to suddenly change during a chemotactic trajectory (*Materials and Methods*). Two policies are trained: one with no sudden changes (*α* = 0), resulting in a persistent policy, and another with frequent reorientations (*α* = 0.3), producing a reactive policy (Fig. 5*A*).

When no sudden change in gradient direction is expected, the resulting configurations further confirm the important role of persistence in accurate chemotaxis, as it enables the cell to polarize (34), and by relying on consecutive turns, the cell further aligns itself slowly with the gradient. In *D. discoideum*, longer times of starvation lead to increased levels of polarization, allowing cells to commit to chemotaxis toward aggregation centers (35). For the policy trained on static environments, at low SNR, two candidate pseudopods with a smaller angular separation than those we constructed from experimental observations, prove optimal, further demonstrating that relying on small changes reduces the likelihood of sudden errors. Given that the limited information the cell gains from the environment is not enough to distinguish forward and side directions, the found policy optimizes for time, which results in a configuration with a minimal number of candidates (*n* = 2), echoing our results on alignment efficiency from Fig. 3*E*. However, as the information from the environment increases, the subtle difference between neighboring candidates becomes apparent. The resulting policy at high SNR activates the forward pseudopod candidate (Fig. 5*A*) while still minimizing the number of candidates to yield better alignment during long trajectories, where the concentration profile remains static. When comparing the CI of the trajectories as a function of the SNR, we obtain an envelope curve (red), resulting in an upper limit to previous performances (Fig. 5*B*).

Training instead with *α* = 0.3, we find, interestingly, that the resulting suppression configurations are less constrained, showing the possibility of activating the rear pseudopod candidates at low SNR and consistently activating them at high SNR, where the frontal candidates are likely to win when the difference in signal is notable (Fig. 5*A*). When evaluating both policies in increasingly dynamic environments (Fig. 5*B*, *Inset*), the performance of the persistent policy rapidly collapses while the reactive policy is only slightly affected by the change, even when persistence is removed in its entirety (α→1).

To evaluate the adaptability of both policies to sudden changes, we calculate the time required for a cell to reorient when the gradient is flipped. To reduce the stochastic effects of random walks, we measure the last passage time, *T*_*L*_, for the cell to cross a line located at a distance *h* behind its initial position (Fig. 5*C*). Interestingly, this analysis reveals a crossover: The persistent configuration reacts more slowly to gradient changes, performing U-turns to realign with the new direction. However, this approach proves more effective than the reactive policy at low SNR (Fig. 5*C*). At high SNR, by contrast, the reactive policy excels, as it can immediately reverse direction when the gradient flips. This behavior is consistent with previous observations (33) and the ability of cells to form de novo pseudopods (11). While these findings align with reported behaviors, experimental validation of such crossings could highlight how the advantages of relying on persistence for enhanced performance can prove costly in more dynamical and fluctuating scenarios. Thus, the advantages of using fewer pseudopod directions that we found in the previous sections vanish as the environments become increasingly dynamic.

## Discussion

We introduced a minimal model for cellular decision-making based on the competition between pseudopods with stimulus-dependent growth. Previously, this inference problem has been considered through an “all-knowing” cell that evaluates the measurements of the chemical gradient to decide its orientation of motion, requiring knowledge of the times and locations of ligand binding events. For instance, Berg and Purcell referred to such a cell as the perfect instrument (22), but did not consider cell motility. In contrast, we propose that pseudopod dynamics, driven by the stochastic processes of actin polymerization, simple G-actin conservation, and mutual inhibition, are responsible for the cell’s emergent decisions. Hence, no direct spatial gradient sensing is required. This is because pseudopod growth just depends on the local attractant concentration, so that the gradient is indirectly inferred from the competition between pseudopods. This has the benefits that no centralized computing is required and that sensing and movement are closely integrated—hallmarks of mechanical intelligence.

Our model captured the key features of pseudopod dynamics as observed in experiments, such as the emergence of two pseudopods in shallow low-SNR gradients for both fast and accurate chemotaxis (9), oscillations in Y-junction microfluidic devices (*SI Appendix*, *Supplement* 6) (20), potentially due to a pitchfork bifurcation underlying the dynamics (19), the scaling of the chemotactic index (CI) with SNR (12), and the scaling of the smallest detectable stimulus (or gradient) with the background concentration, $\Delta c∼c_{0}^{\beta }$, reminiscent of the Weber–Fechner law. Our results, however, reveal that *β* may depend on the number of growth directions, which could provide an explanation for varying values of *β* found across systems (36). This mechanistic explanation reconciles the observed deviations from Weber’s law and provides insight into how cells dynamically adapt their chemotactic sensitivity based on noise levels.

By concatenating consecutive decision steps, i.e., splitting events, we modeled chemotaxis trajectories capable of reproducing experimental data: Our results showed the characteristic dependence of the chemotactic index on the SNR, previously demonstrated in Endres and Wingreen (12), and Tweedy et al. (9). Note that for even higher ligand concentrations, we may expect a decline of the CI due to receptor or second-messenger saturation (21, 28, 29), a trait that our plots also hint to (Fig. 4*D*) given the polymerization rate saturation. We then extended and generalized the model by incorporating a learnable suppression mechanism, allowing us to explore how cells might have optimized their polarization for efficient chemotaxis, particularly at low SNR, where gradient information is especially limited. By employing deep reinforcement learning, we found a mapping between the SNR and pseudopod suppression. Our findings align with experimental observations of a split configuration (11, 37) at low SNR and a frontal, elongated configuration at high SNR (9). Note that the broad front is equivalent to many pseudopods in our model (high *n*).

Our model assumes that cells have a mechanism for suppressing or tailoring certain growth directions based on the SNR. Such a mechanism has previously been described by the Meinhardt reaction–diffusion model (38), for which Neilson et al. (32) and Tweedy et al. (9) showed that a stimulus-dependent activator combined with a local and global inhibitor on a deformable membrane leads to behavior found in real cells, at low and high SNR. While this 3-morphogen model is a coarse-grained representation of complex signaling and cytoskeleton regulating pathways, the morphogens have equivalents in real cells (see *SI Appendix*, *Supplement* 3 for details) (9). Thus, an emergent reaction–diffusion mechanism can lead to SNR-dependent pseudopod patterns as proposed here.

Future experiments could be designed to test our model predictions as follows. First, the pseudopod decision time depends exponentially on the steepness of the gradient (Fig. 2*D*). This makes intuitive sense as easier decisions should be faster. Second, besides cells fulfilling the Weber–Fechner law, we predict that the scaling exponent changes with different background concentration and hence noise regimes, due to different numbers of cell protrusions used (Fig. 3*B*). Third, the turning in switching gradients could be investigated systematically. While such experiments were conducted before (33, 39), the predicted strong dependence on the SNR for different levels of polarization as regulated by starvation (35) were not (Fig. 5*C*). Such an investigation could potentially yield valuable insights into cell mechanisms that rely on persistence to overcome sensing limitations.

Our model also contributes fundamental insight into the physical limits of sensing. Spread-out cell protrusions are optimal for instantaneous sensing, as they increase the spatial information of the environment around the cell (40, 41). Our results suggest that cell polarization promotes forward-facing pseudopods, which, while clearly suboptimal for an instant decision, prove advantageous during chemotaxis. By leveraging persistence, which bypasses the need for a memory of prior information, the cell is capable of maintaining a consistent direction of movement, thereby improving its ability to navigate gradients over longer trajectories. Thus, our findings highlight the tradeoff between instantaneous, accurate sensing and overall chemotactic performance.

Amoeboid cell migration based on pseudopods and other cell protrusions is not unique to *D. discoideum* but also occurs in neutrophils (10) and even in the spermatozoa of *Caenorhabditis elegans* (42). Notably, the latter achieves motility without actin using the major sperm protein (MSP) instead, emphasizing shape and behavior as fundamentally important and that the biochemical details might be secondary (43). Instead, we suggest that amoeboid shape and behavior are critical traits, providing advantages in fast and accurate chemotaxis. Similarly, the syncytial plasmodia of the slime mold *Physarum polycephalum* forages and grows as a macroscopic network (44). Not unlike our proposed stimulus-driven pseudopod extensions, nutrient uptake on one end of the network drives extensions in this favorable direction, leading to retraction at the rear or pruning of side branches of the networks. Key differences, however, include their size, which can reach several centimeters, and the rhythmic contractions of their tubular network, which aid in circulating their internal contents. Nevertheless, strategies for efficient foraging resemble our chemotaxis strategies, involving the cycling between three morphodynamic states (45). Whether our navigation strategies are also relevant to ciliated microorganisms (1) or group chemotaxis (46) are fascinating open questions.

Clearly, our results go beyond chemotaxis in cells. After initial fast actin polymerization, the decision-making between pseudopods occurs on a stereotypical low-dimensional manifold on a slow time scale (19), reminiscent of the log-likelihood ratio test between alternatives in the classic Wald algorithm for optimal sequential decision-making (47). With an increasing interest in developing microscopic artificial agents, a theoretical framework for decision-making in difficult-to-navigate environments is crucial. Amoeba-inspired limbless robotics may benefit from our robust strategy designs without requiring extensive hard-wired sensor-driven feedback mechanisms (48, 49). We showed that by suppressing the strength of candidate directions, the cell can use persistence to effectively navigate up a gradient by focusing on creating fewer forward-facing protrusions.

In conclusion, our work provides insights into the fundamental principles governing cell decision-making and their implications for chemotaxis in complex environments. We showed that pseudopod splitting can facilitate highly effective chemotaxis without a need for direct spatial sensing and memory, suggesting aspects of mechanical intelligence. Additionally, our work illustrates the potential of reinforcement learning as a valuable tool for studying the interplay between cellular mechanics, sensing, and behavior without relying on black-box decision policies that obscure the internal cellular mechanism. These findings may also have applications in robotics.

## Materials and Methods

### Numerical Simulations.

Actin dynamics are integrated using the Euler–Maruyama integration scheme, converging to the Ito solution. Discrete-time steps are set to Δt=0.1 during the simulations unless explicitly stated. Thousands of realizations are carried out for each numerical result by massively parallelizing the simulations using GPUs (*Data, Materials, and Software Availability*). Parameters values used in the simulations are $\rho_{0}=\kappa_{c}=1$, δ=1/3, λ=ϵ=1/2, and $\sigma^{2}=5\times 10^{-4}$. Further details on their estimation can be found in *SI Appendix*, *Supplement* 2.

### Nondimensionalization of the Dynamics.

To simplify the equations, we nondimensionalize the system by scaling all lengths relative to cell size *a*. Similarly, time is defined relative to *τ*_*m*_, a characteristic timescale for the linear filter in Eq. **4**, which relates to the mechanical properties of the membrane. Accordingly, the gradient and concentration levels are rescaled by length scale *a*, resulting in unitless concentration profile. Hence, length scale *a* changes the concentration profile while maintaining the same cell dimensions. In short, the cell dynamics are described in the cell’s frame of reference. Note, our concentrations and gradients match experimental conditions. In the figures presented here, our unitless concentration ranges from 0 to 10^4^ and our gradients from 0 to 20. In contrast, in ref. 21, concentrations and gradients applied in experiments were $10^{-12}$ to $10^{-6}$M and 0 to 25 nM/mm, respectively, which, in our unitless way correspond respectively to 0.6 to $6\times 10^{5}$ and 0 to 150, using length scale *a* = 10 µm.

### Suppression Policy Architecture.

We model the suppression policy *ρ*_*θ*_ as a relatively small artificial neural network whose input is the logarithm in base 10 of the SNR and whose output is a vector of (n,2) values between 0 and 1 as the logits for the probability of activating or suppressing each pseudopod $\rho_{0}$. The final state is sampled from a categorical distribution. The network is a multilayer perceptor (MLP) of 4 layers of 128 neurons each, with tanh activation functions between them. Since the policy also predicts the expected value *V*, we use an MLP with 4 layers and 128 neurons.

### Optimizing the Suppression Policy.

The algorithm used here is a modified version of the PPO algorithm (17) implemented in ref. 4. PPO is an on-policy optimization technique that iteratively improves its policy *p*_*ω*_ by collecting information from simulations between optimization steps. The results of these simulations are then used to perform stochastic gradient descent on the policy parameters *ω*. Simply, the algorithm maximizes the following clipped surrogate loss defined as

$$\begin{array}{c} \begin{array}{cc} L^{\mathrm{CLIP}}\left(\omega \right)= & E[\min(\frac{p_{\omega_{n}}\left(\rho_{0}|z\right)}{p_{\omega_{p}}\left(\rho_{0}|z\right)}\cdot A_{t},\\ & \;\;\text{clip}\left(\frac{p_{\omega_{n}}\left(\rho_{0}|z\right)}{p_{\omega_{p}}\left(\rho_{0}|z\right)},1-\epsilon ,1+\epsilon \right)\times A_{t})], \end{array} \end{array}$$

where *ω*_*n*_ represents the updated parameters of the policy, while *ω*_*p*_ indicates the previous policy parameters. The term $p_{\omega }\left(\rho_{0}|z\right)$ is the probability of output $\rho_{0}$ given the SNR, here indicated by *z*, under the new policy. The advantage function *A*_*t*_ quantifies the relative benefit of taking a particular action at a given state compared to the average action value. Notably, the clipping parameters *ϵ* control the size of the trust region, ensuring that new updates do not deviate significantly from the previous policy, which leads to more stable optimizations. In practice, the clipping is performed by defining upper and lower bounds on the allowed change in ratio between consecutive policies that contribute to the loss.

To promote exploration, especially given the discrete nature of our actions (active or suppressed candidate), we include an entropy term on the loss function as a regularization term set by

$$\begin{array}{c} H=-\frac{1}{n}\sum_{i}^{n}p_{\omega }^{\left(i\right)}\left(z\right)\log\left(p_{\omega }^{\left(i\right)}\left(z\right)\right). \end{array}$$

which encourages the policy to maintain a certain degree of stochasticity, preventing collapses and premature convergence. Here, *n* is the number of actions, which is set as the number of candidate pseudopods.

We define the reward function after each decision step during the chemotactic trajectory of the cell (i) as

[9] $$\begin{array}{c} R^{\left(i\right)}\left(x_{T},T\right)=\cos\left(\theta_{T}\right)+\gamma \left(\frac{t_{\max}-T}{t_{\max}}\right), \end{array}$$

where $t_{\max}$ is the maximum possible time for the cell to make a decision before a random one is selected, and *γ* is a time penalty to favor the configurations that lead to faster events. For this study, we set *γ* = 0.2. Subsequently, we optimize the weights on the network by maximizing the cumulative reward ${\hat{R}}^{\left(i\right)}$ during a trajectory, which we specify as the concatenation of 30 splitting events.

### Simulating Fluctuating Environments.

Chemical environments in nature can be highly dynamical and require cells to be able to react to sudden changes (33). To incorporate this into the simulations, at each splitting event the gradient direction is reoriented with probability *α*, where a new random angle *θ*_*n*_ is chosen from a uniform distribution in [0,2π]. This reorientation mimics a sudden change in gradient direction, disrupting the cell’s ability to rely on persistence from the previous step. Two policies are trained in environments with different *α*: a persistent policy (*α* = 0), in which no reorientation occurs, and a reactive policy (*α* = 0.3), where reorientation can happen. The sudden changes only affect the gradient direction as the background concentration at the FAP remains unchanged.

### Experimental Data.

The experimental frames shown in Fig. 1 *A* and *C* were taken with permission from refs. 10 and 18, respectively. The data points of the chemotactic index used in Fig. 4 were digitized from previous studies (9, 30, 31).

## Supplementary Material

Appendix 01 (PDF)

## Data Availability

All the numerical simulations and deep reinforcement learning implementations are available and can be found at https://github.com/Endres-group/psxc-research (50). All other data are included in the manuscript and/or *SI Appendix*.
