# Supplementary material for: Persistent pseudopod splitting is an effective chemotaxis strategy in shallow gradients
Source: Proc Natl Acad Sci U S A. 2025 May 8;122(19):e2502368122. doi: 10.1073/pnas.2502368122 (PMC12088397; doi:10.1073/pnas.2502368122)
Supplement: Supplementary file 1 — Appendix 01 (PDF) [file pnas.2502368122.sapp.pdf]

PNAS

# Supporting Information for

## Persistent pseudopod splitting is an effective chemotaxis strategy in shallow gradients

**Albert Alonso, Julius B. Kirkegaard and Robert G. Endres**

**Corresponding: Robert G. Endres.**
**E-mail: r.endres@imperial.ac.uk**

### This PDF file includes:

Supporting text
Figs. S1 to S8
SI References

## Supporting Information Text

## Supplement 1

### Gradient sensing by a cell with $N$ sensors

Here, we derive the chemotactic performance of a cell that has surface sensors and can accurately process the information these send to estimate the gradient, also referred to in the main text as an *all-knowing* cell (see Fig. S1). We assume the cell has $N$ sensors in a plane with coordinates $(x_i, y_i)$ for each sensor, with the cell center of mass at the origin. Each sensor measures the ligand concentration $c_i$ with some uncertainty $\delta c_i$.

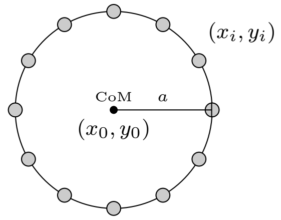


**Fig. S1.** Diagram of a cell with $N$ sensors spread on its surface.

How would the cell infer the direction of the chemical gradient in this plane using least squares fitting?

**Gradient estimation.** Assume the ligand concentration $c$ at any point $(x, y)$ in the plane is given by:

$$c(x, y) = c_0 + g_x x + g_y y \tag{1}$$

where $c_0$ is the concentration at the origin, and $g_x$ and $g_y$ are the components of the gradient vector in the $x$ and $y$ directions, respectively.

Each sensor $i$ located at $(x_i, y_i)$ measures the concentration $c_i$ with uncertainty $\delta c_i$, and the objective is to minimize the sum of squared differences between the measured concentrations and the model concentrations, defined as

$$S = \sum_{i=1}^{N} \left(c_i - (c_0 + g_x x_i + g_y y_i)\right)^2 \tag{2}$$

To minimize it, we take the partial derivatives of $S$ with respect to $c_0$, $g_x$, and $g_y$ and set them to zero:

$$\frac{\partial S}{\partial c_0} = -2 \sum_{i=1}^{N} \left(c_i - (c_0 + g_x x_i + g_y y_i)\right) = 0 \tag{3}$$

$$\frac{\partial S}{\partial g_x} = -2 \sum_{i=1}^{N} x_i \left(c_i - (c_0 + g_x x_i + g_y y_i)\right) = 0 \tag{4}$$

$$\frac{\partial S}{\partial g_y} = -2 \sum_{i=1}^{N} y_i \left(c_i - (c_0 + g_x x_i + g_y y_i)\right) = 0 \tag{5}$$

which can then be rewritten as

$$\sum_{i=1}^{N} c_i = N c_0 + g_x \sum_{i=1}^{N} x_i + g_y \sum_{i=1}^{N} y_i \tag{6}$$

$$\sum_{i=1}^{N} x_i c_i = c_0 \sum_{i=1}^{N} x_i + g_x \sum_{i=1}^{N} x_i^2 + g_y \sum_{i=1}^{N} x_i y_i \tag{7}$$

$$\sum_{i=1}^{N} y_i c_i = c_0 \sum_{i=1}^{N} y_i + g_x \sum_{i=1}^{N} x_i y_i + g_y \sum_{i=1}^{N} y_i^2 \tag{8}$$

and since these are linear equations, we can express them in matrix form as

$$\begin{pmatrix} N & \sum x_i & \sum y_i \\ \sum x_i & \sum x_i^2 & \sum x_i y_i \\ \sum y_i & \sum x_i y_i & \sum y_i^2 \end{pmatrix} \begin{pmatrix} c_0 \\ g_x \\ g_y \end{pmatrix} = \begin{pmatrix} \sum c_i \\ \sum x_i c_i \\ \sum y_i c_i \end{pmatrix} \tag{9}$$

with the gradient estimated direction being $\mathbf{g} = (g_x, g_y)$.

**Simplification for $c_0 = 0$.** If the ligand concentration is measured relative to the center of mass's ligand concentration, effectively making $c_0 = 0$, the problem simplifies as we only need to determine the gradient components $g_x$ and $g_y$. Thus, the model for the concentration at any point $(x_i, y_i)$ changes from (1) to

$$c_i = g_x x_i + g_y y_i \tag{10}$$

with its corresponding objective function

$$S = \sum_{i=1}^{N} \left(c_i - (g_x x_i + g_y y_i)\right)^2 \tag{11}$$

Similarly to before, we take the partial derivatives of $S$ with respect to $g_x$ and $g_y$ and set them to zero:

$$\frac{\partial S}{\partial g_x} = -2 \sum_{i=1}^{N} x_i \left(c_i - (g_x x_i + g_y y_i)\right) = 0 \tag{12}$$

$$\frac{\partial S}{\partial g_y} = -2 \sum_{i=1}^{N} y_i \left(c_i - (g_x x_i + g_y y_i)\right) = 0 \tag{13}$$

which we rewrite and express them in matrix form leading to

$$\begin{pmatrix} \sum_{i=1}^{N} x_i^2 & \sum_{i=1}^{N} x_i y_i \\ \sum_{i=1}^{N} x_i y_i & \sum_{i=1}^{N} y_i^2 \end{pmatrix} \begin{pmatrix} g_x \\ g_y \end{pmatrix} = \begin{pmatrix} \sum_{i=1}^{N} x_i c_i \\ \sum_{i=1}^{N} y_i c_i \end{pmatrix} \tag{14}$$

**Including measurement uncertainty.** So far, we have assumed that the cell can accurately measure the concentration values on its sensors. For a more realistic derivation, we include the measurement errors $\delta c_i$ in the ligand concentrations, which will modify the least squares fitting process to account for the uncertainties in the data. This involves using weighted least squares, where each measurement $c_i$ is weighted based on its uncertainty $\delta c_i$.

In weighted least squares, each residual is weighted by the inverse of the variance of the corresponding measurement. Therefore, the weight for each measurement is $w_i = \frac{1}{\delta c_i^2}$, where $c_i$ is the measured concentration, leading to the objective function:

$$S = \sum_{i=1}^{N} w_i \left(c_i - (g_x x_i + g_y y_i)\right)^2 . \tag{15}$$

Analogously to the previous derivations, we express the linear system in matrix form as

$$\begin{pmatrix} \sum_{i=1}^{N} \frac{x_i^2}{\delta c_i^2} & \sum_{i=1}^{N} \frac{x_i y_i}{\delta c_i^2} \\ \sum_{i=1}^{N} \frac{x_i y_i}{\delta c_i^2} & \sum_{i=1}^{N} \frac{y_i^2}{\delta c_i^2} \end{pmatrix} \begin{pmatrix} g_x \\ g_y \end{pmatrix} = \begin{pmatrix} \sum_{i=1}^{N} \frac{x_i c_i}{\delta c_i^2} \\ \sum_{i=1}^{N} \frac{y_i c_i}{\delta c_i^2} \end{pmatrix} \tag{16}$$

where now the components of the matrices are similar to (14) divided by their uncertainties.

***Solving the system.*** Let the previous linear system (16) be

$$A_w \begin{pmatrix} g_x \\ g_y \end{pmatrix} = \mathbf{b}_w \tag{17}$$

where

$$A_w = \begin{pmatrix} \sum_{i=1}^{N} \frac{x_i^2}{\delta c_i^2} & \sum_{i=1}^{N} \frac{x_i y_i}{\delta c_i^2} \\ \sum_{i=1}^{N} \frac{x_i y_i}{\delta c_i^2} & \sum_{i=1}^{N} \frac{y_i^2}{\delta c_i^2} \end{pmatrix}; \quad \mathbf{b}_w = \begin{pmatrix} \sum_{i=1}^{N} \frac{x_i c_i}{\delta c_i^2} \\ \sum_{i=1}^{N} \frac{y_i c_i}{\delta c_i^2} \end{pmatrix} \tag{18}$$

the inverse of $A_w$ is given by

$$A_w^{-1} = \frac{1}{\det(A_w)} \begin{pmatrix} A_{22} & -A_{12} \\ -A_{21} & A_{11} \end{pmatrix} \tag{19}$$

where $\det(A_w)$ is the determinant of $A_w$:

$$\det(A_w) = A_{11}A_{22} - A_{12}A_{21} \tag{20}$$

Using the elements of $A_w$ from (17):

$$A_{11} = \sum_{i=1}^{N} \frac{x_i^2}{\delta c_i^2}, \quad A_{12} = A_{21} = \sum_{i=1}^{N} \frac{x_i y_i}{\delta c_i^2}, \quad A_{22} = \sum_{i=1}^{N} \frac{y_i^2}{\delta c_i^2} \tag{21}$$

we can express (19) as

$$A_w^{-1} = \frac{1}{\left(\sum_{i=1}^N \frac{x_i^2}{\delta c_i^2}\right)\left(\sum_{i=1}^N \frac{y_i^2}{\delta c_i^2}\right) - \left(\sum_{i=1}^N \frac{x_i y_i}{\delta c_i^2}\right)^2} \begin{pmatrix} \sum_{i=1}^N \frac{y_i^2}{\delta c_i^2} & -\sum_{i=1}^N \frac{x_i y_i}{\delta c_i^2} \\ -\sum_{i=1}^N \frac{x_i y_i}{\delta c_i^2} & \sum_{i=1}^N \frac{x_i^2}{\delta c_i^2} \end{pmatrix}, \quad [22]$$

The gradient components $g_x$ and $g_y$ are obtained by multiplying $A_w^{-1}$ with $\mathbf{b}_w$:

$$\begin{pmatrix} g_x \\ g_y \end{pmatrix} = A_w^{-1}\mathbf{b}_w \quad [23]$$

which we use (22) and (17) to express as

$$\begin{pmatrix} g_x \\ g_y \end{pmatrix} = \frac{1}{\det(A_w)} \begin{pmatrix} \sum_{i=1}^N \frac{y_i^2}{\delta c_i^2} & -\sum_{i=1}^N \frac{x_i y_i}{\delta c_i^2} \\ -\sum_{i=1}^N \frac{x_i y_i}{\delta c_i^2} & \sum_{i=1}^N \frac{x_i^2}{\delta c_i^2} \end{pmatrix} \begin{pmatrix} \sum_{i=1}^N \frac{x_i c_i}{\delta c_i^2} \\ \sum_{i=1}^N \frac{y_i c_i}{\delta c_i^2} \end{pmatrix} \quad [24]$$

with

$$\det(A_w) = \left(\sum_{i=1}^N \frac{x_i^2}{\delta c_i^2}\right)\left(\sum_{i=1}^N \frac{y_i^2}{\delta c_i^2}\right) - \left(\sum_{i=1}^N \frac{x_i y_i}{\delta c_i^2}\right)^2 \quad [25]$$

Performing the matrix multiplication and simplifying the results leads to

$$g_x = \frac{\sum_{i=1}^N \frac{y_i^2}{\delta c_i^2} \sum_{i=1}^N \frac{x_i c_i}{\delta c_i^2} - \sum_{i=1}^N \frac{x_i y_i}{\delta c_i^2} \sum_{i=1}^N \frac{y_i c_i}{\delta c_i^2}}{\left(\sum_{i=1}^N \frac{x_i^2}{\delta c_i^2}\right)\left(\sum_{i=1}^N \frac{y_i^2}{\delta c_i^2}\right) - \left(\sum_{i=1}^N \frac{x_i y_i}{\delta c_i^2}\right)^2} \quad [26]$$

$$g_y = \frac{\sum_{i=1}^N \frac{x_i^2}{\delta c_i^2} \sum_{i=1}^N \frac{y_i c_i}{\delta c_i^2} - \sum_{i=1}^N \frac{x_i y_i}{\delta c_i^2} \sum_{i=1}^N \frac{x_i c_i}{\delta c_i^2}}{\left(\sum_{i=1}^N \frac{x_i^2}{\delta c_i^2}\right)\left(\sum_{i=1}^N \frac{y_i^2}{\delta c_i^2}\right) - \left(\sum_{i=1}^N \frac{x_i y_i}{\delta c_i^2}\right)^2} \quad [27]$$

These formulas provide the gradient estimation components $g_x$ and $g_y$, accounting for the uncertainties in the measurements.

**Uncertainty in gradient estimation.** The Cramer-Rao bound (CRB) provides a lower bound on the variance of any unbiased estimator (1–3). For our problem, we can derive the CRB to quantify the uncertainty in the gradient measurement $\delta\mathbf{g}$.

To derive the CRB, we first need to compute the Fisher information matrix (FIM). For a set of measurements $\{c_i\}$ with uncertainties $\{\delta c_i\}$ and the simplified linear profile (10), the FIM is given by:

$$\mathcal{I}(\mathbf{g}) = \sum_{i=1}^N \frac{1}{\delta c_i^2} \begin{pmatrix} x_i^2 & x_i y_i \\ x_i y_i & y_i^2 \end{pmatrix} \quad [28]$$

which we reformulate it as

$$\mathcal{I}(\mathbf{g}) = \begin{pmatrix} I_{xx} & I_{xy} \\ I_{xy} & I_{yy} \end{pmatrix}, \quad [29]$$

where

$$I_{xx} = \sum_{i=1}^N \frac{x_i^2}{\delta c_i^2}, \quad I_{xy} = \sum_{i=1}^N \frac{x_i y_i}{\delta c_i^2}, \quad I_{yy} = \sum_{i=1}^N \frac{y_i^2}{\delta c_i^2} \quad [30]$$

The CRB states that the covariance matrix of any unbiased estimator $\hat{\mathbf{g}}$ is bounded from below by the inverse of the FIM. The covariance matrix of $\hat{\mathbf{g}} = (g_x, g_y)^T$ is

$$\mathrm{Cov}(\hat{\mathbf{g}}) \geq \mathcal{I}(\mathbf{g})^{-1} \quad [31]$$

To find $\mathcal{I}(\mathbf{g})^{-1}$, we compute the inverse of the 2x2 matrix $\mathcal{I}(\mathbf{g})$:

$$\mathcal{I}(\mathbf{g})^{-1} = \frac{1}{I_{xx}I_{yy} - I_{xy}^2} \begin{pmatrix} I_{yy} & -I_{xy} \\ -I_{xy} & I_{xx} \end{pmatrix}, \quad [32]$$

Hence, the variances (uncertainties) of $g_x$ and $g_y$ are the diagonal elements of $\mathcal{I}(\mathbf{g})^{-1}$:

$$(\delta g_x)^2 \geq \left(\mathcal{I}(\mathbf{g})^{-1}\right)_{11} = \frac{I_{yy}}{I_{xx}I_{yy} - I_{xy}^2} \quad [33]$$

$$(\delta g_y)^2 \geq \left(\mathcal{I}(\mathbf{g})^{-1}\right)_{22} = \frac{I_{xx}}{I_{xx}I_{yy} - I_{xy}^2} \quad [34]$$

substituting (30) into (33), we obtain the standard deviation to be

$$\delta g_x = \sqrt{\frac{\sum_{i=1}^N \frac{y_i^2}{\delta c_i^2}}{\left(\sum_{i=1}^N \frac{x_i^2}{\delta c_i^2}\right)\left(\sum_{i=1}^N \frac{y_i^2}{\delta c_i^2}\right) - \left(\sum_{i=1}^N \frac{x_i y_i}{\delta c_i^2}\right)^2}} \quad [35]$$

$$\delta g_y = \sqrt{\frac{\sum_{i=1}^N \frac{x_i^2}{\delta c_i^2}}{\left(\sum_{i=1}^N \frac{x_i^2}{\delta c_i^2}\right)\left(\sum_{i=1}^N \frac{y_i^2}{\delta c_i^2}\right) - \left(\sum_{i=1}^N \frac{x_i y_i}{\delta c_i^2}\right)^2}} \quad [36]$$

These expressions provide the lower bounds on the standard deviations of the estimates of $g_x$ and $g_y$, taking into account the uncertainties in the ligand concentration measurements.

**Simplified formulas for radial symmetry.** The resulting equations become cumbersome, but due to the symmetries of our system, we can simplify them.

Assuming the cell sensors sit on a circle of radius $a$ such that $x_i^2 + y_i^2 = a^2$ (see Fig. S1), we have

$$\sum_{i=1}^{N}(x_i^2 + y_i^2) = Na^2 \quad [37]$$

which, due to the sensors being symmetrically distributed, we also obtain the following relations

$$\sum_{i=1}^{N} x_i^2 = \sum_{i=1}^{N} y_i^2 = \frac{Na^2}{2} \quad [38]$$

$$\sum_{i=1}^{N} x_i y_i = 0 \quad [39]$$

Furthermore, we assume that the uncertainty of measuring concentrations is the same for all sensors $\delta c_i = \delta_c$, which leads to

$$\sum_{i=1}^{N} \frac{x_i^2}{\delta c_i^2} = \frac{1}{\delta c^2}\sum_{i=1}^{N} x_i^2 = \frac{1}{\delta c^2}\frac{Na^2}{2} \quad [40]$$

with this, we can simplify (26-27) to

$$g_x = \frac{2}{Na^2}\sum_{i=1}^{N} x_i c_i \quad [41]$$

$$g_y = \frac{2}{Na^2}\sum_{i=1}^{N} y_i c_i \quad [42]$$

and similarly, the uncertainties (35-36) to

$$\delta g_x = \sigma_g = \sqrt{\frac{2\delta c^2}{Na^2}} \quad [43]$$

$$\delta g_y = \sigma_g = \sqrt{\frac{2\delta c^2}{Na^2}} \quad [44]$$

**Chemotactic Index (CI).** Based on the derived gradient estimation, we can determine the expected chemotactic index (CI) of a cell that, at each step, decides to move toward the estimated gradient direction. The CI is defined as the mean cosine similarity of the direction of movement and the true gradient direction, which we can calculate with

$$CI = \int\int_{-\infty}^{\infty} \frac{\hat{\mathbf{g}}\cdot\mathbf{g}}{\|\hat{\mathbf{g}}\|\|\mathbf{g}\|} P(\hat{g}_x \hat{g}_y) d\hat{g}_x d\hat{g}_y, \quad [45]$$

where $\hat{\mathbf{g}}$ is the estimated gradient and $\mathbf{g}$ is the real gradient, where we assume the estimated gradients are Gaussian-distributed such that the probability is

$$P(\hat{g}_x, \hat{g}_y) = \frac{1}{\sqrt{2\pi}\delta g_x}\exp\left[\frac{-(\hat{g}_x - g_x)^2}{2\delta g_x^2}\right]\frac{1}{\sqrt{2\pi}\delta g_y}\exp\left[\frac{-(\hat{g}_y - g_y)^2}{2\delta g_y^2}\right], \quad [46]$$

Furthermore, assuming the real gradient to only be on the $x$-direction, the dot product simplifies to:

$$\hat{\mathbf{g}} \cdot \mathbf{g} = \hat{g}_x \cdot g_x + \hat{g}_y \cdot 0 = \hat{g}_x \cdot g_x \qquad [47]$$

with magnitudes

$$\|\hat{\mathbf{g}}\| = \sqrt{\hat{g}_x^2 + \hat{g}_y^2}, \quad \|\mathbf{g}\| = g_x \qquad [48]$$

by including (46), (47) and (48) to (45) we obtain

$$CI = \int \int_{-\infty}^{\infty} \frac{\hat{g}_x g_x}{\sqrt{\hat{g}_x^2 + \hat{g}_y^2}} \frac{1}{\sqrt{2\pi}\delta g_x} \exp\left(\frac{-(\hat{g}_x - g_x)^2}{2\delta g_x^2}\right) \frac{1}{\sqrt{2\pi}\delta g_y} \exp\left(\frac{-(\hat{g}_y)^2}{2\delta g_y^2}\right) d\hat{g}_x d\hat{g}_y \qquad [49]$$

Despite its apparent complexity, we can solve this integral analogously to Endres and Wingreen (4) and obtain

$$\text{CI} = \sqrt{\frac{\pi z}{2}} e^{-z} \left[I_0(z) + I_1(z)\right], \qquad [50]$$

where $I_{0(1)}$ are first (second)-order modified Bessel functions, and, differently to the derivation using the physical limits of sensing, our $z$ is given by

$$z = \frac{Na^2}{8} \frac{g_x^2}{\delta c^2} = \frac{Na^2 k 5\pi}{24} \text{SNR} \qquad [51]$$

where we have use the uncertainty $\delta c$ expression from Berg and Purcell (5) for the error in measurement

$$\delta c = \frac{3c_0}{5\pi DT}. \qquad [52]$$

A comparison plot with the pseudopod splitting model and the fundamental limit from the main text is provided in Fig. S2.

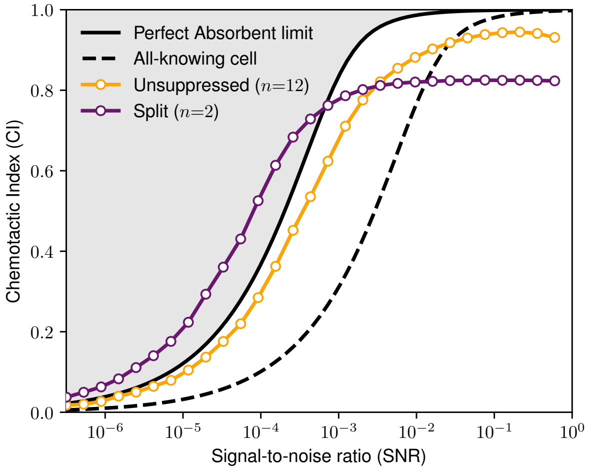


**Fig. S2. Chemotactic index (CI) of an *all-knowing* cell.** Results from using Eq. (51) in Eq. (50) with the same constant value as used in the perfect absorbing cell, alongside the CI for the simulated configurations with our mechanical model. As shown here, the all-knowing cell performs below the perfect absorber limit, and is thus less relevant for describing CI data.

## Supplement 2

### Simulation parameters

Here, we provide a list of the parameters used in our decision-making model based on stimulus-dependent growth and competition between pseudopods for recruiting actin. While we used generic non-dimensional parameters of order 1 for simplicity, the results are fairly robust to parameter changes (see below for examples of parameter variation). A similar approach was recently used in Ron et al. (6). Note also that while our parameters used in simulations are generic, the strategies discovered are confirmed by optimization from machine learning, indicating robustness of our results. Nevertheless, the parameters can, in principle, be motivated or derived from observation.

We used cell length $a$ and averaging time of actin fluctuations $\tau_m$ as our length and time units for nondimensionalization. Cell length for *Dictyostelium discoideum* (Dicty) amoeba and neutrophils of our immune system is about 10µm (7) and $9-15$µm (8), respectively.

The averaging time for the actin dynamics, $\tau_m$, relates to the mechanical properties of the membrane. By comparing the mean of the decision time $T_D$ ($\sim 6$), which is the growth time of a losing pseudopod, or mean of the total duration $T$ ($\sim 8$), which is the growth time of the winning pseudopod, in Fig. 2c of the main text to the mean of the distribution of measured pseudopod growth times ($\sim 8s$), we can infer $\tau_m \sim$ 1s (9, 10). As pseudopods are formed continuously, our durations $T_D$ or $T$ translate into $< 10$ pseudopods per minute, which compares favorably with the measured number of $\sim 4$ (11). The measured pseudopod tip speed of about $\sim 0.5\mu$m/s (9, 11) can be converted to our unitless model by considering that $\tau_m \sim$ 1s and $a = 10\mu$m. However, $\rho_0$ in Eq. 3 in the main text, is the actin polymerization rate without any length dependence, and the pseudopod growth rate is a resulting emergent property many processes in our model (Fig. 2 in the main text).

The susceptibility of the receptor free energy $\kappa_c \sim 1/K_D$ is approximately the inverse ligand dissociation constant $K_D$ of the receptors, which is in the range of $10-100$nM for Dicty (cAMP) (12, 13) and $\sim 10\mu$M for neutrophils (chemokine CXCL1) (14).

The noise parameter $\sigma$ is really propagated external Berg & Purcell-type ligand noise (5), causing polymerized actin levels in pseudopods to fluctuate. To understand this better we illustrate this with a toy model of a receptor with occupancy $n$ and pseudopod F-actin level $A$:

$$\frac{dn}{dt} = k_+ c(1-n) - k_- n \qquad [53]$$

$$\frac{dA}{dt} = k_p n A_u - k_d A, \qquad [54]$$

where $A_u = 1 - A$ for uncommitted monomeric actin, using linear actin dynamics for simplicity. The total actin noise is then

$$\sigma^2_{A,\mathrm{tot}} = \sigma^2_{A,\mathrm{int}} + \left(\frac{\tau_c}{\tau_A}\right)\sigma^2_c \qquad [55]$$

$$= 2k_d A + \left(\frac{k_d}{DaTk_-}\right)c, \qquad [56]$$

where the first intrinsic noise term is estimated assuming small Poissonian noise around steady state (15). The second term describes time-averaged extrinsic Berg-and-Purcell-type ligand noise ($\sigma^2_c \sim c/(DaT)$) (5, 16). For simplicity we only consider extrinsic noise in our model in the main text with $\sigma^2 \sim k_d/(DaTk_-)$.

The remaining parameters such as exchange rate $\epsilon$ and cross-inhibition $\lambda$ are compound parameters, emerging from the interplay of many molecular species confined inside the cell membrane. These can in principle be fitted by matching higher-level statistical data such as fitting the drift speed, persistence, Weber-Fechner law, or mean-squared displacement to experimental data (17, 18), or by mapping out phase diagrams of different cell behaviors (19). Regarding the former, we found that $\Delta c = kc^\beta$ where the scaling parameter $k$ is determined by the value of $\epsilon$, while the exponent $\beta$ is influenced by the number of pseudopods (see main text Fig. 3b and supplementary Fig. S7).

## Supplement 3

## Connection to previous models and molecular species

We have assumed that cells have a mechanism for suppressing or tailoring certain growth directions based on the SNR. Such a mechanism has previously been described by the Meinhardt reaction-diffusion model (20), for which Neilson et al. (21) and Tweedy et al. (22) showed that a stimulus-dependent activator combined with a local and global inhibitor on a deformable membrane leads to behavior found in real cells. At low SNR, cells predominantly split pseudopods while at high SNR, cells have a broad front (corresponding to multiple, active pseudopods in our model) (22). Pseudopods are autonomously formed and removed in cycles: the activator leads to pseudopod formation, the local inhibitor splits and removes them subsequently, and the global inhibitor makes additional pseudopods harder to form. This works for evenly distributed receptors, in line with experimental observation (23). While this 3-morphogen model is a coarse-grained representation of complex signaling and cytoskeleton regulating pathways, they have equivalents in real cells (22). The local activator could potentially be ARP2/3 complex, initiating the branching (and hence growth) of actin filaments, or PI3-K recruiting to the leading edge of cells to stimulate the conversion of PIP2 to PIP3. Coronin could play the role of the local inhibitor, known to inhibit actin filament nucleation and observed to co-localise with F-actin. cGMP may act as a global inhibitor. It diffuses quickly and so its concentration would quickly equilibrate across the cell. Depletion of G-actin or even membrane tension may also provide an effective global inhibition mechanism, through long-range competition between pseudopods. Thus, an emergent Turing-like mechanism can lead to SNR-dependent pseudopod patterns as proposed here.

## Supplement 4

### Decision-time dependency on chemoattractant profile

In our mechanistic model of the main text (Fig. 2d), there is a dependency of the decision-making time on the environmental gradient and background concentration. In more detail, we observe a clear exponential decay as the gradient is increased (until saturation). This is validated by fitting to function $T_D = \hat{T} \cdot \exp(-\gamma \cdot g) + T_D^*$, plotted as the logarithm of $T_D - T_D^*$ in Fig. S3. However, we observe that all the fitted values show a dependency on the background concentration (Fig. S3).

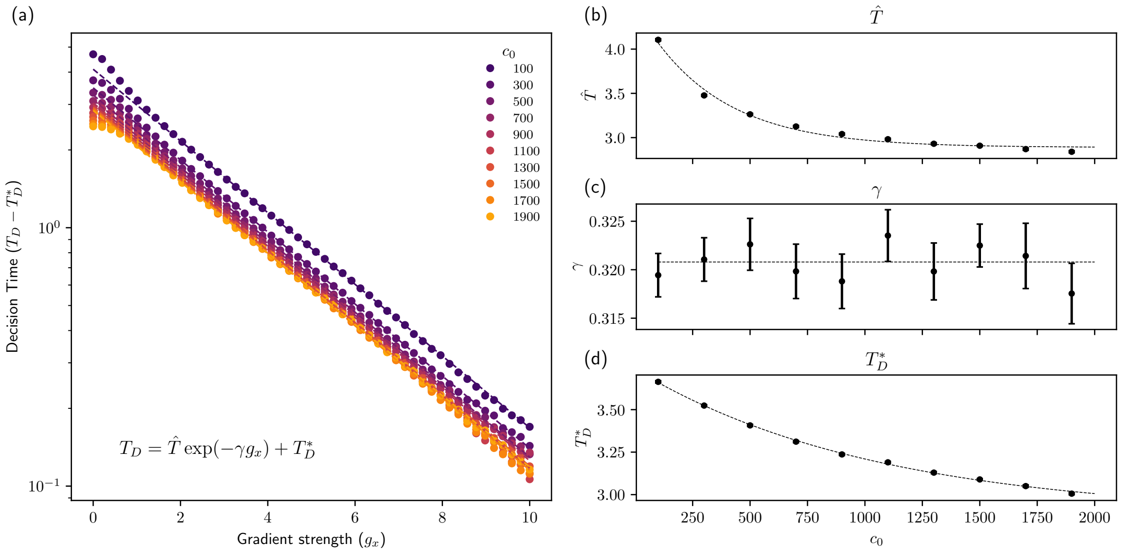


**Fig. S3. Decision time dependency on gradient strength ($g_x$) and background concentration ($c_0$). (a)** Log-linear plot of decision time ($T_D - T_D^*$) as a function of gradient strength $g_x$, illustrating the exponential dependence. The data for various background concentrations $c_0$ (from 100 to 1900) are fitted to the model $T_D = \hat{T}\exp(-\gamma g_x) + T_D^*$. **(b)** Estimated $\hat{T}$ as a function of $c_0$, showing a decreasing trend. **(c)** Fitted $\gamma$ values for different $c_0$, indicating no significant dependence on background concentration. **(d)** Saturating decision time $T_D^*$ as a function of $c_0$, showing an exponential decrease with increasing background concentration.

Based on these results, noise accelerates decision-making in our model, leading to faster decisions. However, as shown in other sections of the main text, this speed comes at a cost: higher noise levels reduce the accuracy of the decisions. To better understand this interplay, we analyze the combined effects of gradient strength ($g_x$) and background concentration ($c_0$) on accuracy and decision time (Fig. S4).

Interestingly, we find that both increasing $g_x$ and increasing $c_0$ result in shorter decision times, but through fundamentally different mechanisms. A stronger gradient ($g_x$) reduces the decision time while simultaneously improving accuracy, since it provides a clearer signal for the decision-making process (Fig. S4c, inset). In contrast, increasing background concentration ($c_0$) reduces the decision time by amplifying noise, but at the expense of reduced accuracy (Fig. S4c). Thus, our simple illustrative model exhibits non-trivial behavior, displaying the trade-offs between speed and accuracy in distinct ways depending on the interplay between signal and noise.

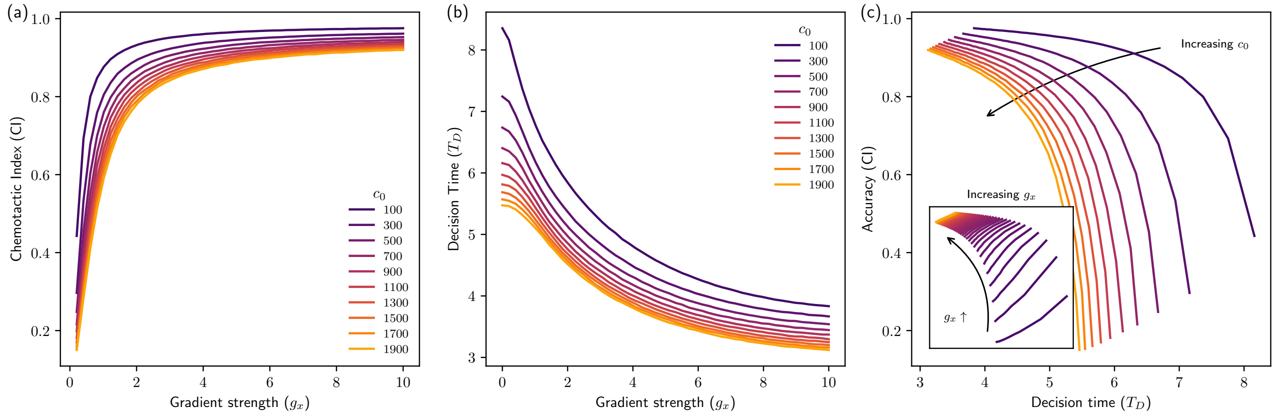


**Fig. S4. Effect of gradient strength ($g_x$) and background concentration ($c_0$) on chemotactic accuracy and decision-making time.** **(a)** Chemotactic index (CI) as a function of gradient strength ($g_x$) for various background concentrations ($c_0$). Higher background concentrations result in a slower increase in CI with increasing gradient strength, indicating reduced accuracy in noisy environments. **(b)** Decision time ($T_D$) as a function of gradient strength ($g_x$) for different background concentrations ($c_0$). Decision time decreases with increasing gradient strength and is consistently shorter for higher background concentrations. **(c)** Accuracy (CI) versus decision time ($T_D$) across different values of $g_x$ and $c_0$. Each curve corresponds to a fixed background concentration, with increasing $g_x$ leading to improved accuracy and reduced decision time. The inset highlights the trend, where each line corresponds to the same $g_x$ and they are colored by $g_x$ strength. Likewise, it shows that lower $g_x$ leads to lower accuracy and longer decision times, particularly in high-noise environments. Noticeable is that while increasing both properties (gradient and concentration) reduces the decision time, one points towards an increase in accuracy whereas the other tends to a decrease.

## Supplement 5

### Parameter robustness on decision making dynamics

This section analyzes the robustness of actin competition dynamics to parameter variations (Fig. S5). While the exchange parameter $\varepsilon$ exerts the strongest control over decision speed (oscillatory indecision at $\varepsilon \to 0$ vs rapid commitment at $\varepsilon \gg 0$), the system remains stable across most tested ranges of the rest of parameters. Deviations emerge only at extreme parameter values, demonstrating that core decision-making features persist under variation of parameter values.

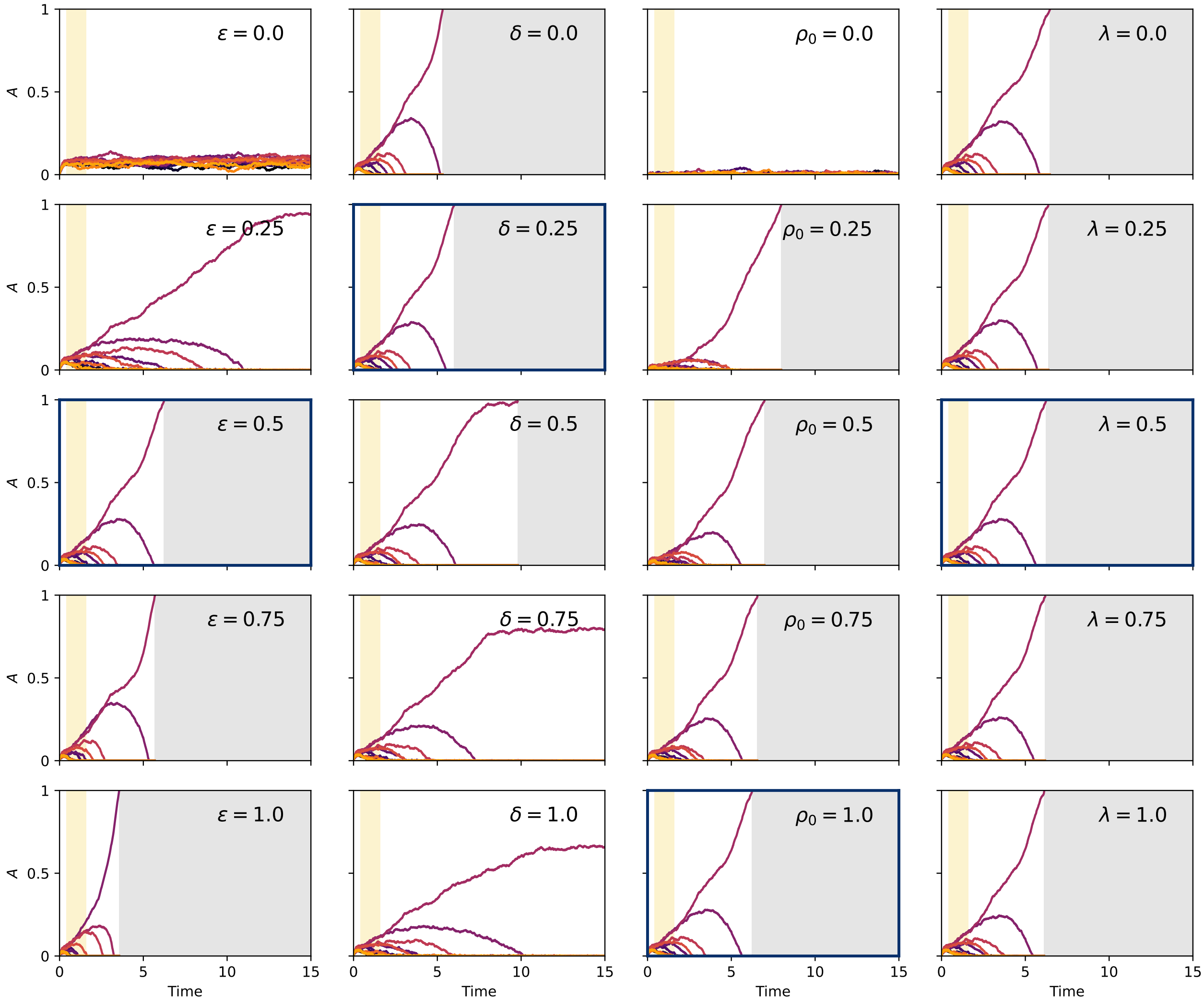


**Fig. S5. Robustness check on decision-making dynamics.** Simulation results of F-actin dynamics in a system of $n = 12$ candidate pseudopods at gradient $g_x = 10$ and background concentration $c_0 = 10^3$, for varying values of key simulation parameters: $\varepsilon$, $\delta$, $\rho_0$, and $\lambda$. The normalized F-actin levels $(A)$ are shown over time for each parameter set, allowing a direct comparison of their influence on the pseudopod competition dynamics. The exchange parameter $\varepsilon$ (first column) has the most pronounced effect, as increasing $\varepsilon$ promotes faster and more decisive competition dynamics. For small $\varepsilon$, the system exhibits persistent oscillatory dynamics, while larger $\varepsilon$ values lead to rapid convergence, where a single pseudopod dominates. The parameter $\delta$ (second column), which governs the sensitivity of pseudopods to inhibitory feedback, shows a similar trend. Lower $\delta$ values reduce the duration of competition, favoring quick resolution, whereas higher values result in prolonged periods of indecision. The initial activation parameter $\rho_0$ (third column) affects the initial distribution of F-actin levels. Higher values of $\rho_0$ lead to steeper growth curves, but the overall dynamics remain consistent with the default case, except at the extremes where competition becomes more abrupt. Lastly, the parameter $\lambda$ (fourth column), which encodes the strength of cross-inhibition appears to, albeit showcasing some effect, have less influence over the dynamics than the other parameters. The blue-highlighted panels correspond to parameter values near the default simulation parameters, which represent a biologically plausible baseline. From this comparison, we find that the system is robust to small perturbations in most parameters, with deviations only occurring at extreme values. The shaded regions in the plots represent different stages of the decision-making process: decision-making (yellow), growth (white) and event finished (gray). Parameters such as $\kappa_c$ and $\sigma^2$ are excluded here, as their effects are equivalent to altering the signal-to-noise ratio (SNR) and are analyzed extensively elsewhere in the manuscript.

## Supplement 6

### Modeling fluctuating Y-junction scenarios

This section demonstrates how our decision-making framework can capture key features of fluctuating pseudopod competition in three-way (or Y) junctions, as experimentally observed by Ron et al. (6). By setting $\varepsilon \leq 0.2$ (no strong *commit-to-the-winner* mechanism), we reproduce the oscillatory dynamics they describe, where no single pseudopod stabilizes as a winner. This aligns with their observations of exploratory fluctuations in symmetrical environments.

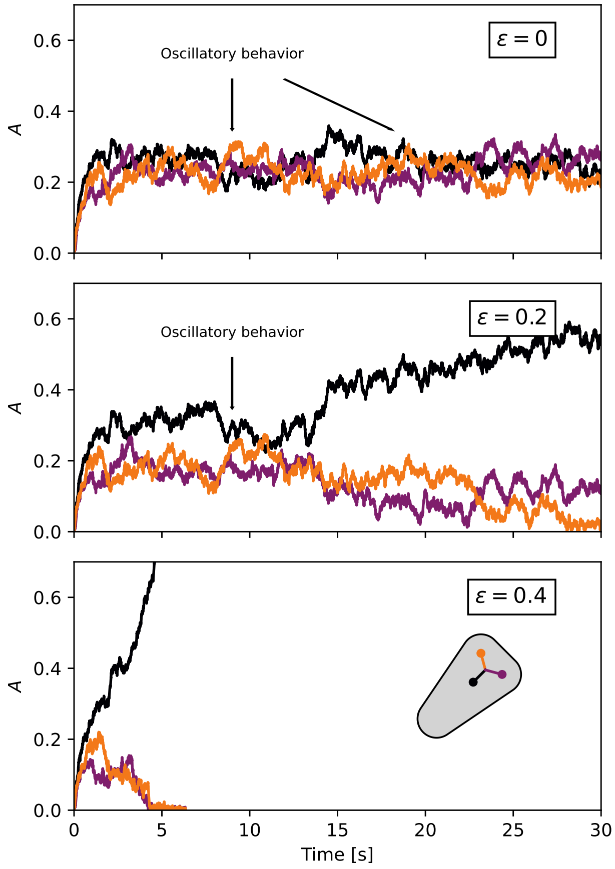


**Fig. S6. Simulation results of F-actin dynamics in a microfluidic Y-junction device.** Here, three pseudopods are positioned equidistantly ($n = 3$), as described in the recent work by Ron et al. (6). The panels illustrate the temporal evolution of normalized F-actin levels ($A$) in the absence of external signaling ($g_x = 0$), under varying values of the exchange parameter $\varepsilon$. This parameter encodes a *commit-to-the-winner* mechanism, in which the pseudopod with higher F-actin levels suppresses the growth of competing pseudopods, thereby facilitating robust decision-making. For $\varepsilon = 0$, the model reproduces the observed oscillatory dynamics (6), demonstrating the generality and consistency of our approach. As $\varepsilon$ increases to $\varepsilon = 0.25$ and $\varepsilon = 0.5$, the oscillatory behavior diminishes. For higher values of $\varepsilon$, the system transitions to a regime dominated by rapid decision-making, with a clear suppression of competing pseudopods shortly after initialization. The shaded region in the $\varepsilon = 0.5$ panel highlights the rapid selection process. The diagram in the lower-right corner of the $\varepsilon = 0.5$ panel illustrates the Y-junction pseudopod configuration, with pseudopod directions color-coded to match the corresponding traces in the time series plots. These findings underscore the influence of $\varepsilon$ in controlling the balance between oscillatory exploration and decisive commitment in pseudopod competition. The results could be further explored under various external signaling conditions to assess broader biological implications and extend the discoveries of Ron et al. (6), e.g. to X junctions.

## Supplement 7

### Threshold of minimum scaling can be set by $\varepsilon$

Even though the resulting dynamics are rather robust to parameter perturbations (Fig. S5), there are situations in which changing them alters the displayed values. For instance, the exchange rate parameter ($\varepsilon$) can change the constant ($G$) used to describe the minimum stimulus scaling in the Weber-Fechner law (see the main text), set by

$$\bar{g} = G \cdot c_0^{\beta}, \qquad [57]$$

while the scaling $\beta$ itself is not affected (unless the value changes enough that the dynamics break), as seen in Fig. S7. This demonstrates that the reported scaling is a fundamental behavior of the system and is not merely a result of the specific parameter values.

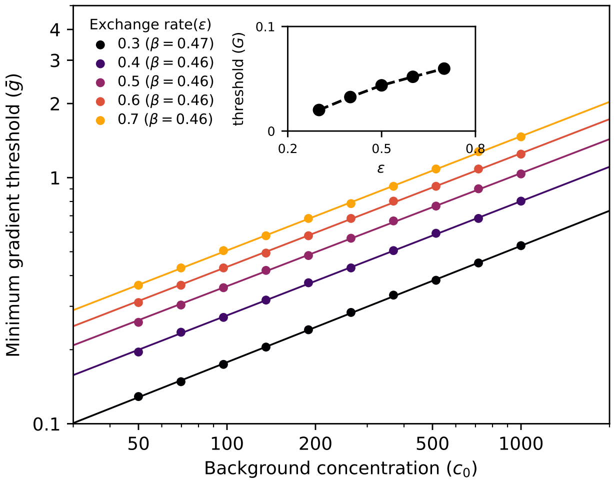


**Fig. S7. Dependence of the minimum gradient threshold $\bar{g}$ on the background concentration $c_0$ for different values of the exchange rate parameter $\varepsilon$, for a system of $n = 6$ candidate pseudopods.** The relationship $\bar{g} = G \cdot c_0^{\beta}$ is illustrated, where the scaling exponent $\beta$ remains constant $\beta = 0.46$) across all conditions – although it slightly changes when the dynamics start to break at $\varepsilon$=0.3. The exchange rate $\varepsilon$ affects the constant $G$, as evidenced by the vertically shifted lines corresponding to different $\varepsilon$ values (0.3, 0.4, 0.5, 0.6, and 0.7). Larger $\varepsilon$ leads to higher $G$, resulting in elevated gradient thresholds for a given $c_0$. The inset shows a linear trend between $\varepsilon$ and $G$, demonstrating how $G$ increases systematically with the exchange rate. These findings illustrate that while the scaling exponent $\beta$ remains robust, the parameter $G$ is sensitive to changes in $\varepsilon$, altering the required minimum stimulus scaling without affecting the underlying scaling law.

## Supplement 8

## Exponential filtering of actin dynamics

To further test the robustness of our results, we replace the linear time-averaging filter in Eq. (4) of the main text with an exponential kernel characterized by a decay time $\tau$. The exponential filtering is defined as:

$$\ell_i(t) = L \int_{-\infty}^{t} A_i(t') e^{-(t-t')/\tau} dt', \tag{58}$$

where $\tau = 1$ is chosen to match the characteristic time scale of the linear filter for comparison. As shown in Fig. S8, the results with exponential filtering are nearly identical to those obtained with the linear filter. This consistency demonstrates that the choice of kernel does not qualitatively affect the predictions of our model.

Although exponential filtering is biologically intuitive because of its ease of implementation in cellular processes, we use the linear kernel in the main text for simplicity in mathematical expression and analysis. The supplementary plot compares the chemotactic index (CI) across a range of signal-to-noise ratios (SNRs) for both filtering approaches, confirming their equivalence in outcome in one of the major results of the main text.

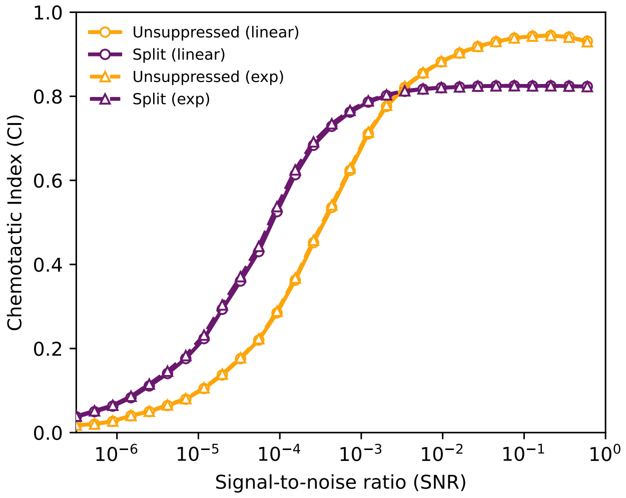


**Fig. S8. Comparison of linear and exponential filters for actin dynamics.** Here, the chemotactic index (CI) is shown as a function of signal-to-noise ratio (SNR), plotted on a $\log_{10}$ scale. The yellow curves represent the CI calculated for the unsuppressed case, where all pseudopods are active, while the purple curves correspond to the split configuration, where only a subset of pseudopods (e.g., candidates $\mathcal{P}_3$ and $\mathcal{P}_{11}$) are activated. Circular markers indicate results using the linear filter (Eq. (4), main text), and triangular markers represent results obtained with the exponential filter (Eq. (58)). Both filtering approaches yield nearly identical results, confirming the robustness of the model to the choice of filter. The exponential kernel is biologically intuitive due to its ease of implementation in cellular processes, but the linear kernel is used in the main text for simplicity in mathematical expression. This plot includes fewer entries compared to the main text to emphasize the comparison between filtering approaches.

## References


1. G Aquino, NS Wingreen, RG Endres, Know the single-receptor sensing limit? think again. *J. Stat. Phys.* **162**, 1353–1364 (2016).
2. B Hu, W Chen, WJ Rappel, H Levine, How geometry and internal bias affect the accuracy of eukaryotic gradient sensing. *Phys. Rev. E* **83**, 021917 (2011).
3. A Hopkins, BA Camley, Chemotaxis in uncertain environments: hedging bets with multiple receptor types. *Phys. Rev. Res.* **2**, 043146 (2020).
4. RG Endres, NS Wingreen, Accuracy of direct gradient sensing by single cells. *Proc. Natl. Acad. Sci.* **105**, 15749–15754 (2008).
5. HC Berg, EM Purcell, Physics of chemoreception. *Biophys. J.* **20**, 193–219 (1977).
6. JE Ron, et al., Emergent seesaw oscillations during cellular directional decision-making. *Nat. Phys.* **20**, 501–511 (2024).
7. F Rivero, et al., The role of the cortical cytoskeleton: F-actin crosslinking proteins protect against osmotic stress, ensure cell size, cell shape and motility, and contribute to phagocytosis and development. *J. Cell Sci.* **109**, 2679–2691 (1996).
8. J Prinyakupt, C Pluempitiwiriyawej, Segmentation of white blood cells and comparison of cell morphology by linear and naïve Bayes classifiers. *BioMedical Eng. OnLine* **14**, 63 (2015).
9. L Bosgraaf, PJV Haastert, Quimp3, an automated pseudopod-tracking algorithm. *Cell Adhesion & Migr.* **4**, 46–55 (2010).
10. PJMv Haastert, Unified control of amoeboid pseudopod extension in multiple organisms by branched f-actin in the front and parallel f-actin/myosin in the cortex. *PLOS ONE* **15**, e0243442 (2020).
11. PJM van Haastert, Short- and long-term memory of moving amoeboid cells. *PLOS ONE* **16**, 1–32 (2021).
12. PR Fisher, R Merkl, G Gerisch, Quantitative analysis of cell motility and chemotaxis in dictyostelium discoideum by using an image processing system and a novel chemotaxis chamber providing stationary chemical gradients. *J. Cell Biol.* **108**, 973–984 (1989).
13. PJM van Haastert, M Postma, Biased random walk by stochastic fluctuations of chemoattractant-receptor interactions at the lower limit of detection. *Biophys. J.* **93**, 1787–1796 (2007).
14. KV Sawant, et al., Chemokine CXCL1 mediated neutrophil recruitment: Role of glycosaminoglycan interactions. *Sci. Reports* **6**, 33123 (2016).
15. M Thattai, A Oudenaarden, Attenuation of noise in ultrasensitive signaling cascades. *Biophys. J.* **82**, 2943–2950 (2002).
16. J Paulsson, Summing up the noise in gene networks. *Nature* **427**, 415–418 (2004).
17. PJMV Haastert, A model for a correlated random walk based on the ordered extension of pseudopodia. *PLOS Comput. Biol.* **6**, e1000874 (2010).
18. PJ Van Haastert, A stochastic model for chemotaxis based on the ordered extension of pseudopods. *Biophys. J.* **99**, 3345–3354 (year?).
19. D Pais, et al., A mechanism for value-sensitive decision-making. *PLOS ONE* **8**, e73216 (2013).
20. H Meinhardt, Orientation of chemotactic cells and growth cones: models and mechanisms. *J. Cell Sci.* **112**, 2867–2874 (1999).
21. MP Neilson, et al., Chemotaxis: a feedback-based computational model robustly predicts multiple aspects of real cell behaviour. *PLOS Biol.* **9**, e1000618 (2011).
22. L Tweedy, B Meier, J Stephan, D Heinrich, RG Endres, Distinct cell shapes determine accurate chemotaxis. *Sci. Reports* **3**, 2606 (2013).
23. M Ueda, T Shibata, Stochastic signal processing and transduction in chemotactic response of eukaryotic cells. *Biophys. J.* **93**, 11–20 (2007).
